# The Small, Slow and Specialized CRISPR and Anti-CRISPR of *Escherichia* and *Salmonella*

**DOI:** 10.1371/journal.pone.0011126

**Published:** 2010-06-15

**Authors:** Marie Touchon, Eduardo P. C. Rocha

**Affiliations:** 1 Institut Pasteur, Microbial Evolutionary Genomics, Département Génomes et Génétique, Paris, France; 2 CNRS, URA2171, Paris, France; 3 UPMC Université Pierre et Marie Curie, Atelier de Bioinformatique, Paris, France; Yale University, United States of America

## Abstract

Prokaryotes thrive in spite of the vast number and diversity of their viruses. This partly results from the evolution of mechanisms to inactivate or silence the action of exogenous DNA. Among these, Clustered Regularly Interspaced Short Palindromic Repeats (CRISPR) are unique in providing adaptive immunity against elements with high local resemblance to genomes of previously infecting agents. Here, we analyze the CRISPR loci of 51 complete genomes of *Escherichia* and *Salmonella*. CRISPR are in two pairs of loci in *Escherichia*, one single pair in *Salmonella*, each pair showing a similar turnover rate, repeat sequence and putative linkage to a common set of *cas* genes. Yet, phylogeny shows that CRISPR and associated *cas* genes have different evolutionary histories, the latter being frequently exchanged or lost. In our set, one CRISPR pair seems specialized in plasmids often matching genes coding for the replication, conjugation and antirestriction machinery. Strikingly, this pair also matches the cognate *cas* genes in which case these genes are absent. The unexpectedly high conservation of this anti-CRISPR suggests selection to counteract the invasion of mobile elements containing functional CRISPR/*cas* systems. There are few spacers in most CRISPR, which rarely match genomes of known phages. Furthermore, we found that strains divergent less than 250 thousand years ago show virtually identical CRISPR. The lack of congruence between *cas*, CRISPR and the species phylogeny and the slow pace of CRISPR change make CRISPR poor epidemiological markers in enterobacteria. All these observations are at odds with the expectedly abundant and dynamic repertoire of spacers in an immune system aiming at protecting bacteria from phages. Since we observe purifying selection for the maintenance of CRISPR these results suggest that alternative evolutionary roles for CRISPR remain to be uncovered.

## Introduction

Prokaryotic viruses (phages) are the most abundant forms of life on Earth [1]. Nevertheless, microbes routinely survive and thrive in remarkably phage-rich environments [2]. This is because bacteria and archaea have developed defense mechanisms that allow them to withstand viral predation and the constant exposure to exogenous nucleic acids such as prevention of adsorption, blocking of injection, and abortive infection. Other defense systems do not specifically target phages but any incoming DNA, and include restriction modification systems (RMS) and the use of sugar-nonspecific nucleases [3]. Recently, an adaptive microbial immune system, clustered regularly interspaced short palindromic repeats (CRISPR) has been identified that provides acquired immunity against any foreign DNA by targeting nucleic acid in a sequence-specific manner [4], [5], [6], [7].

CRISPR have been identified in most archaeal (∼90%) and many bacterial (∼40%) genomes thus far sequenced [8], [9], [10]. CRISPR typically consist of short (23–47 bp) and highly conserved direct repeats regularly separated by stretches of variable sequences called spacers. Twelve majors groups of CRISPR were defined based on sequence similarity of their repeats and their ability to form stable RNA secondary structures [11]. CRISPR are often adjacent to *cas* (CRISPR-associated) genes. Cas proteins carry functional domains typical of nucleases, helicases, polymerases, and polynucleotide-binding proteins, involved in the propagation and functioning of CRISPR [12], [13]. They were classified into eight CRISPR/*cas* subtypes that often share gene order as well as content [12]. CRISPRs are typically preceded by an AT-rich non-coding sequence conserved within but not between species called “leader” [8]. A new repeat-spacer unit is added to the CRISPR between the leader and the previous unit, which suggests this particular sequence is likely to include a binding site for the proteins (probably Cas proteins) responsible for repeat duplication and/or spacer acquisition. The leader has also been proposed to act as a promoter for the transcription of the repeat-spacer array into a CRISPR transcript, the pre-crRNA [14], [15]. A fully functional CRISPR/*cas* system is composed of the CRISPR, the Cas proteins and the leader sequence.

Previous studies have reported that many spacers of CRISPR derive from sub-sequences, named proto-spacers, of foreign genetic elements, such as viruses and plasmids [13], [16], [17], [18], [19]. It has therefore been hypothesized that CRISPR/*cas* might be immunity-like systems. This role was first shown experimentally in 2007 in *Streptococcus thermophilus*: CRISPR-harboring strains became resistant to infection by phages after the acquisition of new spacers derived from the virus [4]. More recently, a decreased sensitivity to lambda phage has been reported for *E. coli* strains carrying artificial CRISPR/*cas* systems with spacer targeting essential gene of the virus [20]. It has also been shown that CRISPR/*cas* systems can limit plasmid conjugation in *Staphylococcus epidermidis* [21], demonstrating a broader role for CRISPR in the prevention of horizontal gene transfer (HGT).

While details of CRISPR functioning remain mysterious, it probably involves several steps: (i) CRISPR expression, the transcription of the poly-spacer precursor crRNA, which is followed by binding to a complex of Cas proteins and processing to mono-spacer crRNAs that serve as the guide sequences; and (ii) CRISPR interference, the binding and/or degradation of the target nucleic acid (DNA); (iii) CRISPR modification by the insertion of novel specificity determinants (spacers). Here, we investigate the structure and evolution of CRISPR in 51 complete genomes of *Escherichia* and *Salmonella*. These two genera include important pathogens and model bacteria with highly dynamic genomes [22]. There is also substantial information for mobile genetic elements in these genera. Here, we aim at understanding the evolutionary history of CRISPR and its association with mobile elements in a phylogenetic framework. For this we analyze 51 genomes of three species and two genus, *Escherichia* and *Salmonella*, and relate the structure of the CRISPR with a set of phylogenetic analysis. While we were making the final changes to this manuscript an interesting complementary work was published [23]. This report analyzed only *E. coli* strains without a phylogenetic framework, but used a much larger number of strains than our work (100) and includes brief descriptions of some interesting experimental work. Our results are largely concordant, in what respects *E. coli*, but our phylogenetic analyses leads to some very different conclusions regarding the evolution of CRISPR, as described in the Discussion section.

## Results and Discussion

### Four CRISPR but only two patterns of change

**Figure 1:**
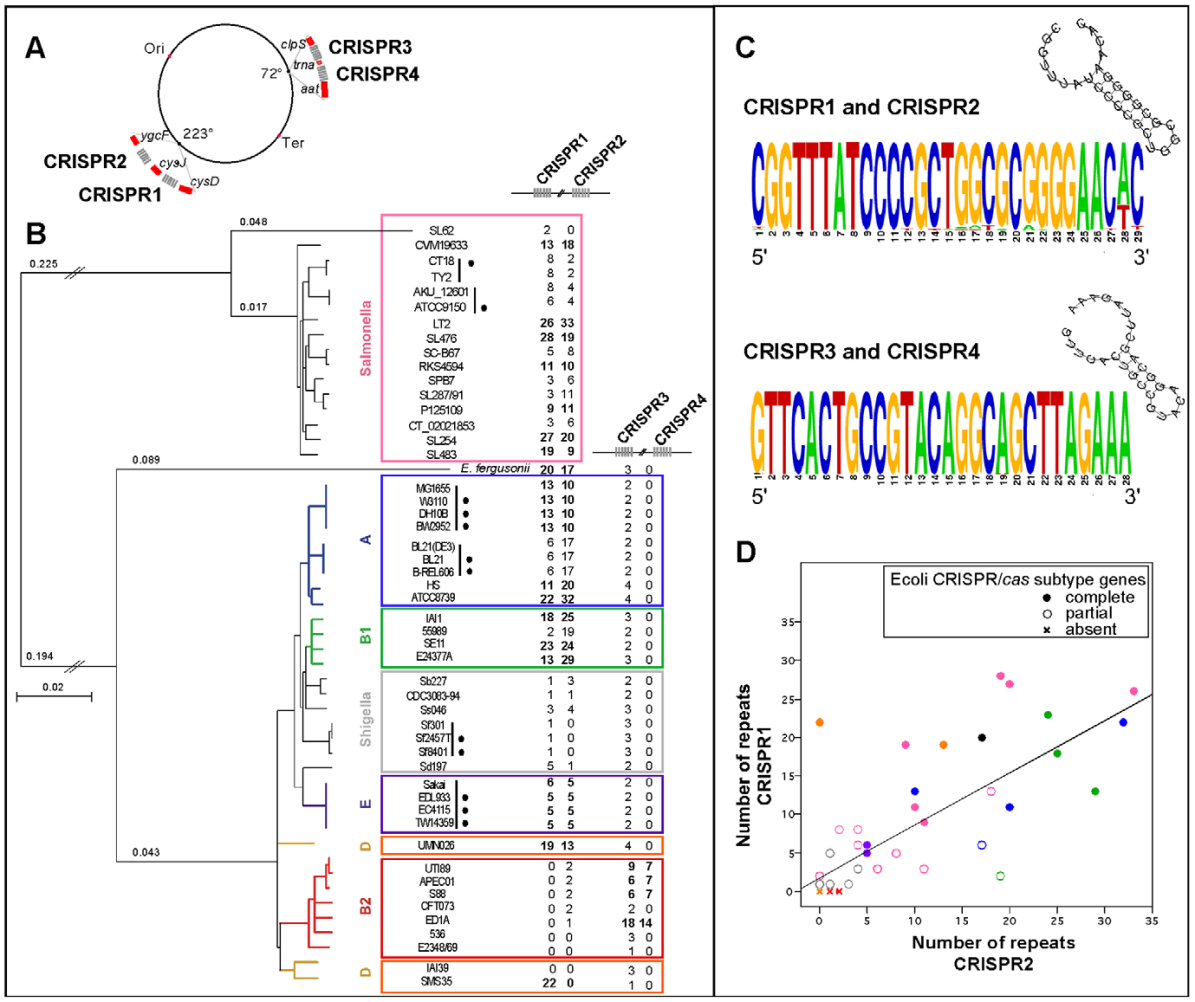
General features of the 4 distinct CRISPR loci. (A) - Position of the 4 distinct CRISPR loci in the chromosomes. Core genes are represented in red; CRISPR in grey. (B) Evolution of the number of repeats of each CRISPR locus across the phylogenetic tree of the 27 *E. coli*, 7 *Shigella*, 16 *Salmonella*, and 1 *E. fergusonii* strains. The tree was reconstructed from the concatenated alignments of 1241 genes of the core genome of *Escherichia* and *Salmonella* strains (see Methods). The main nodes of these branches were supported with high bootstrap values (>90%). Phylogenetic groups of the strains are indicated with colors on the right part of the figure. Very closely related genomes, at distances lower than 0.02% substitutions per position are indicated by a vertical black line; black circles correspond to genomes removed of some analysis, as marked in the text, to avoid redundancy. (C) Sequence logo for all but terminal repeats of the CRISPR1-CRISPR2 arrays and those of the CRISPR3-CRISPR4 arrays. Predicted secondary structure of the most frequent sequence within each CRISPR pairs using RNAfold (see Methods). (D) Positive correlation between the number of repeats in the CRISPR1 and those of the CRISPR2 in each strain (R^2^ = 0.63; p<0.0001). Phylogenetic group of the strains is indicated with colors (see (B)); full circle: CRISPR/*cas* system is complete in the strain; open circle: the system is partial; cross: the system is absent.

We identified 125 CRISPR among the 51 genomes of *Escherichia* and *Salmonella*. Two distinct CRISPR were found in most *Salmonella* chromosomes, and three in most *Escherichia* chromosomes. These 125 CRISPR are located in only 4 distinct loci relative to the *Salmonella/Escherichia* core genome (Figure 1A; see Materials and Methods). The first locus (called CRISPR1) is located between the core genes *cysD-cysJ* and correspond to the first CRISPR array described in the literature [24]. The second (CRISPR2) is located between the core genes *cysJ-ygcF*, distant by less than 20 kb of the CRISPR1 locus. These two loci are jointly detected in all genomic sequences investigated, with the exception of some *Escherichia* genomes belonging to the B2 group and some *Shigella* (Figure 1B). The two other loci (called CRISPR3 and CRISPR4) are also close to each other (<9kbp) located between the core genes *clpS-aat*. They are separated by a tRNA_Ser_ gene present even in genomes lacking these CRISPR, suggesting it predates the creation of the CRISPR4. These CRISPR are absent in *Salmonella*. CRISPR3 is present in all genomes of *Escherichia*, while CRISPR4 is only present in genomes of the B2 group suggesting it is a very recent creation/acquisition. Interestingly, all genomes carrying CRISPR4 lack CRISPR1. As a result, no genome in our set has more than 3 CRISPR.

We defined the typical repeat sequence as the most frequent sequence within a particular CRISPR locus. Few deviations exist to these sequences (Figure 1C), with the exception of the terminal repeat that is almost always degenerated at its 3′ end [8] (Table S5). Remarkably, among the 1053 repeats detected, only two typical repeat sequences coexist (Figure 1C). CRISPR1 and CRISPR2 have the same repeat sequence of 29 bp, while the two other loci have one very different, but also identical and highly conserved, repeat of 28bp (Table 1). These repeats belong respectively to the repeat cluster 2 and 4 previously defined [11]. These two repeats are partially palindromic, having the potential to form stable RNA secondary structures (Figure 1C). Although the presence and number of CRISPR loci is relatively constant, the number of repeats in each locus is highly variable between strains. Two extreme behaviors are observed: either the number of repeats is extremely low (e.g. 1 or 2) or it is relatively high (up to 33) (Figure 1B). This result probably reflects the functionality of the system; highly active systems might have more repeats and higher turnover of spacers. This is detailed below. Interestingly, we observed a strong positive correlation between the number of repeats in the CRISPR1 locus and those of the CRISPR2 locus (R^2^ = 0.63; p<0.0001) (Figure 1D). The same association seems to prevail between CRISPR3 and CRISPR4 loci, but in this case it is based on the analysis of only 4 genomes (the ones containing CRISPR4). There is no positive correlation between the number of repeats in CRISPR1+CRISPR2 and CRISPR3+CRISPR4, indicating that the effect is not general to genomes or due to phylogenetic inertia but specific to the loci. These results suggest that there are two functionally distinct CRISPR pairs in these genomes. CRISPR1 and CRISPR2 on one hand and CRISPR3 and CRISPR4 on the other: these pairs are co-localized in the genome, they have identical repeats and they tend to show similar dynamics.

**Table 1:** Characteristics of the repeat arrays.

	CRISPR1	CRISPR2	CRISPR3	CRISPR4
Flanking core genes	cysD-cysJ	cysJ-ygcF	clpS-aat	clpS-aat
Consensus Repeat sequence	Repeat1	Repeat1	Repeat2	Repeat2
Size Repeat (bp)	29	29	28	28
Free energy of the folded RNA sequences	−14.73 kcal/mol		−8.74kcal/mol	
Number of repeats	433	471	114	35
Exact consensus repeat (terminal repeats were removed)	84% (329/390)	75% (292/390)	97% (77/79)	97% (30/31)
Modified terminal repeat	86%	100%	100%	100%
Number of genomes	43 (84%)	43 (84%)	35 (69%)	4 (8%)
Number of repeats per genome (min-max)	1–28	1–33	1–18	7–14
Number of genomes w/both	38 (74%)		4 (8%)	
Mean distance between each CRISPR pair (kbp)	20 (9−>28)		9	

Repeat1: CGGTTTATCCCCGCTGGCGCGGGGAAC(A/T)C.

Repeat2: GTTCACTGCCGTACAGGCAGCTTAGAAA.

### Only one CRISPR/cas system but different subtypes and frequent transfer

**Figure 2:**
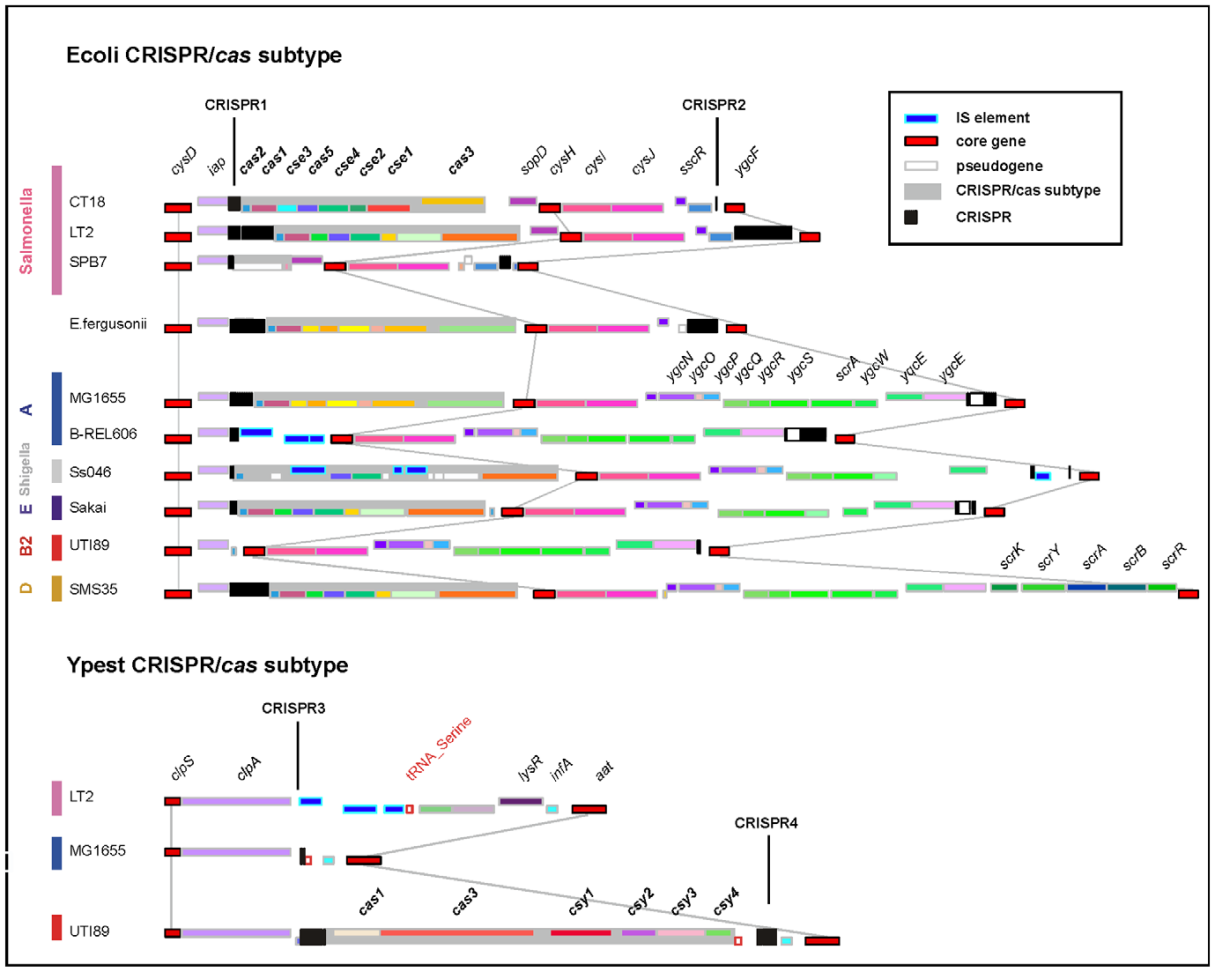
Locus architecture and gene organization for 13 representative CRISPR-associated genomic regions. Ecoli CRISPR/*cas* subtype is characterized by the presence of 8 successive co-oriented genes: *cas2*, *cas1*, *cse3*, *cas5*, *cse4*, *cse2*, *cse1* and *cas3*. Ypest CRISPR/*cas* subtype is characterized by the presence of 6 co-oriented genes: *cas1*, *cas3*, *csy1*, *csy2*, *csy3* and *csy4*. Repeat-spacer arrays are shown as black boxes. Homologous genes are represented using an identical color scheme. Homologous genes were defined by identifying unique pairwise reciprocal best hits, with at least 60% similarity in amino acid sequence and less than 20% of difference in protein length. IS elements appear in blue; pseudogenes in white; and core genes in red. Grey rectangles represent CRISPR/*cas* genes system. A thin gray line connects core genes.

To assess CRISPR functionality we identified the *cas* genes, their location and their characteristics (see Methods, Tables S1–S2). Ecoli CRISPR/*cas* subtype genes appear exclusively on one side of the CRISPR1 arrays. This subtype is characterized by the presence of 8 successive co-oriented genes (Figure 2). We found 23 seemingly complete and 17 partial (e.g. with pseudogenes) such systems in *Salmonella* and *Escherichia* genomes. We found no trace of genes with homology to the Ecoli CRISPR/*cas* subtype in the genomes without the CRISPR1 array. We detected Ypest CRISPR/*cas* subtype in the 4 genomes containing both the CRISPR3 and CRISPR4 and lacking the CRISPR1 array. These two CRISPR arrays flank the 6 genes of the *cas*, but CRISPR4 is separated from the *cas* system by a tRNA_Ser_ gene (Figure 2). Thus, the *cas* genes are always co-localized with a CRISPR array, there is no occurrence of two *cas* systems in the same genome of our set, albeit [23] finds one such occurrence among 100 strains, and the different *cas* systems are associated with the two different families of CRISPR. This is in agreement with the correspondence between the CRISPR/*cas* subtypes and repeat sequences arrays [6], [11]. It is also in agreement with the proposed functional linkage within the two pairs of CRISPR.

**Figure 3:**
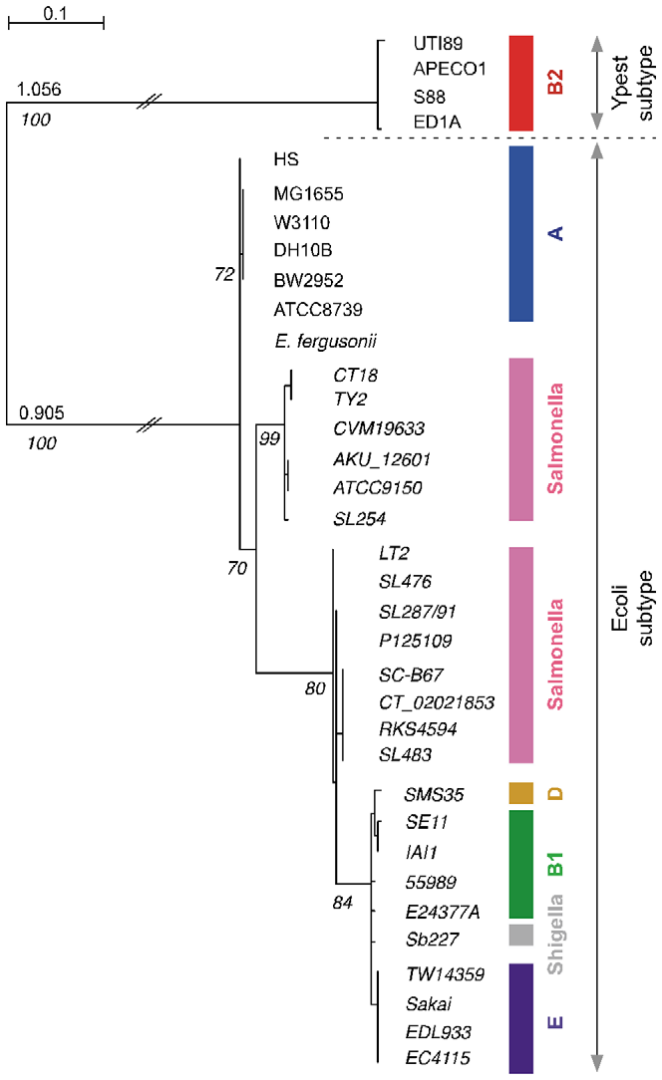
Molecular phylogeny of the Cas1 protein across 35 *Escherichia*/*Salmonella*. Phylogenetic tree for the Cas1 proteins was performed using PhyML with the WAG+G model [49]. Phylogenetic group and CRISPR/*cas* system subtype belonging of the strains are indicated with colors and with arrows respectively on the right part of the figure. Values correspond to aLRT values.

We then explored the molecular phylogeny of Cas proteins. A representative tree for Cas1, which is considered the universal marker of CRISPR-associated systems [9], shows that each subtype forms a monophyletic group (Figure 3). Among the Ecoli CRISPR/*cas* subtype, there are 4 distinct groups, which are incongruent with the phylogenetic relationships of the complete genomes (Figure 1). The phylogeny of *cas* tends to follow the species phylogeny within these 4 groups. Thus, *cas1* has been transferred a few times but after transfer it is transmitted in an essentially vertical way. Trees for the other Cas proteins showed largely the same pattern (data not shown), indicating co-transfer of the entire group of *cas* genes. This hypothesis is further supported by the analysis of the Ypest CRISPR/*cas* subtype. The complete system is present in phylogenetically distant chromosomes and in one plasmid (see Methods, Table S3). These results are consistent with previous reports on the high transmissibility of CRISPR and their association with plasmids, megaplasmids, and even prophages [9]. Yet, it raises an intriguing question: while the systems were several times transferred, they were always transferred to the same genomic location next to one of the CRISPR locus. This suggests that the CRISPR-associated genomic regions are «hot spots» of recombination for elements carrying *cas* elements. This might result from homologous recombination events. Yet, the divergence between most *cas* genes is too high to allow homologous recombination in the *cas* genes [25]. Instead recombination might have taken place at flanking conserved sequences (e.g. at the CRISPR) thereby leading to the replacement of the entire *cas* locus, as proposed for *E. coli* hotspots [26]. With this data one cannot know whether such recombination events replaced *cas* genes or re-introduced them after loss.

### Degraded CRISPR/cas loci show frequent loss of the system

The latter scenario is compatible with multiple events of gain and loss of *cas* genes. Indeed, we find evidence of multiple ongoing processes of CRISPR locus degradation by total or partial deletions in the *cas* genes cluster (Table S1). Examples include both *cas3* and *cse1* pseudogenes adjacent to apparently intact Ecoli CRISPR/*cas* subtype in 9 of the 16 *Salmonella* genomes (allowing subtype identification) (Figure 2). In *Salmonella enterica* SL92, SPB7 and in the 3 strains of *Shigella flexneri*, the Ecoli CRISPR/*cas* subtype is deleted and only a *cas3* pseudogene is still present. *Shigella* exhibit the most dramatic degradation of Ecoli CRISPR/*cas* systems which contain frameshifts, truncations, and IS insertions in almost all the *cas* genes (Figure 2). This is consistent with the ongoing degradation of *Shigella* genomes associated with lower effective population sizes, high abundance of transposable elements and pseudogenes [27], [28], [29]. This result might also reflect a lower prevalence of phage infections in *Shigella* [23]. However, the other genomes showing *cas* degradation do not have appreciably different numbers of prophages in their genomes.

The number of repeats in a CRISPR depends on the level of decay of the associated *cas* genes. The number of repeats is high when the *cas* system is complete, intermediate when the erosion of the system is recent and reduced to a few copies when only relics of the system are detectable (Figure 1D). Interestingly, 3 very close *E. coli* genomes (i.e. BL21-DE3, BL21 and B-REL606) have a large number of repeats while being devoid of *cas* genes. The presence of IS elements on both sides of the CRISPR-associated genomic regions supports the hypothesis of a recent complete deletion of the *cas* system in the latter (Figure 2). In *E.coli* strain SMS35 a complete absence of CRISPR2 was detected due to a recent insertion of a sucrose operon (Figure 2). In general, the number of repeats is a good indicator of the integrity, thus possibly the functionality, of the system.

The analysis of *cas* genes presence/absence and the length of CRISPR gives further support for the linkage between pairs of CRISPR systems. The Ecoli CRISPR/*cas* subtype is always present on one side of the CRISPR1 array, but both CRISPR1 and CRISPR2 arrays are larger when *cas* genes are present next to CRISPR1 (Figure 1D). These results strongly suggest that CRISPR/*cas* act in trans on CRISPR arrays with identical repeat sequences. Thus, the CRISPR2 could be seen as a satellite CRISPR of the CRISPR1 locus, even though it is separated from the latter by several genes of the core genome. The CRISPR3 and CRISPR4 are present on both sides of the Ypest CRISPR/*cas* subtype and also appear to be functionally coupled. We propose that the primary CRISPR correspond to the CRISPR array located directly near the *cas* genes cluster, in these cases CRISPR1 and CRISPR3. Overall, these results suggest that *cas* genes are frequently lost and gained, not simply replaced. They also suggest that CRISPR might out-live the *cas* genes in the genome, thereby providing for an integration hotspot. Finally, they suggest that periods of *cas*-activity in the genome are associated with increase in CRISPR arrays and that the remaining periods are associated with the loss of spacers.

### Leader sequences may or may not be shared/conserved

**Figure 4:**
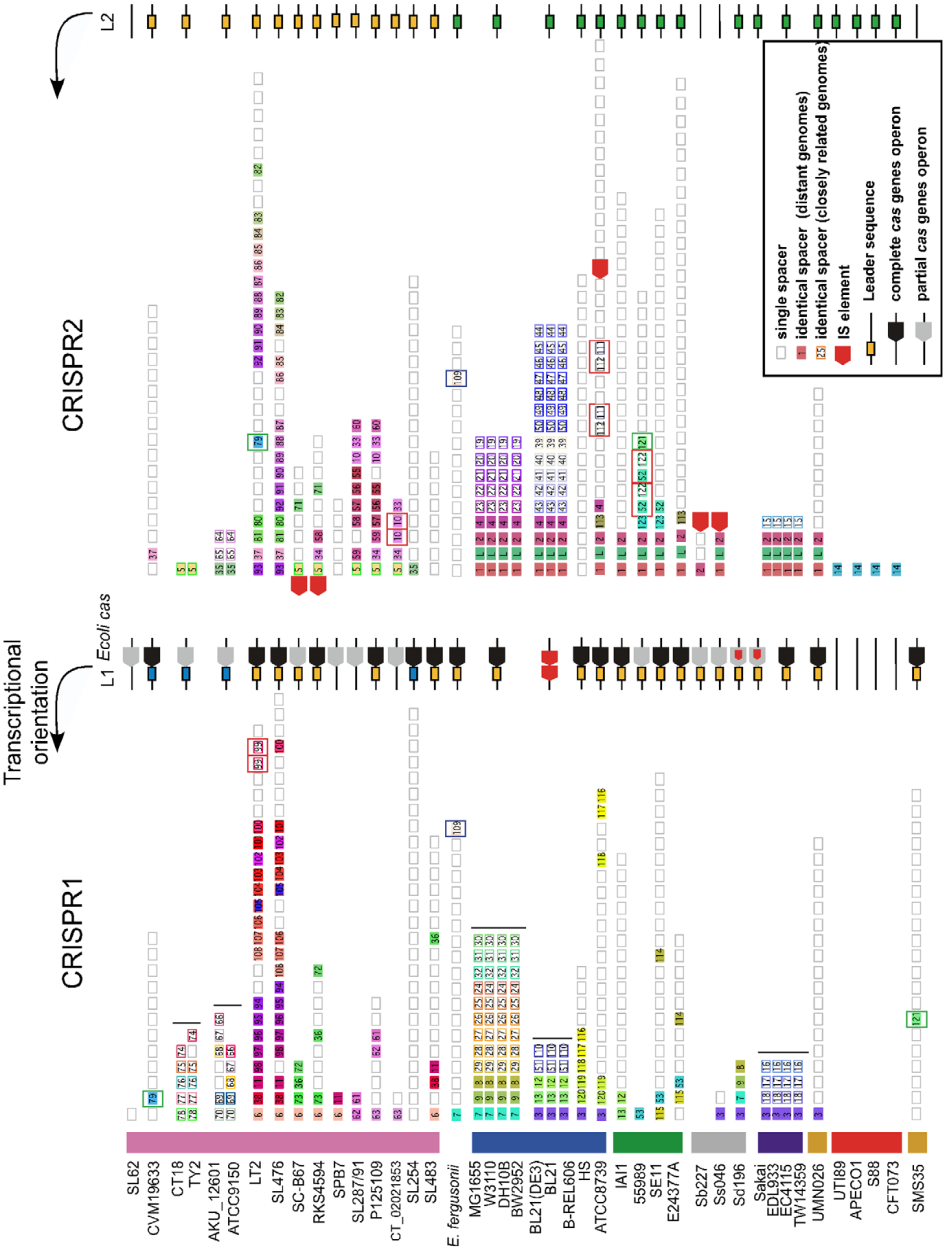
Graphic representation of spacers across CRISPR1 and CRISPR2 in all genomes analyzed. Repeats are not included; only spacer are shown as grey boxes; single spacers appear in white background; identical spacers are represented using a similar color background scheme and identical number. Strains phylogenetic groups are indicated with colors on the left part of the figure. Very closely related genomes (<0.02% substitutions per position) are indicated by a vertical black line. In these cases, identical spacers between these very closely related genomes but single when only one of these strains was considered are represented using a similar color border and number with a white background. Duplication events within the same CRISPR are represented by red boxes, those between CRISPR by green boxes. Similar leaders (called L1 and L2) are represented using identical color background scheme (i.e. yellow, green and blue). IS elements appear as red arrows; complete Ecoli CRISPR/cas subtypes are represented using a black arrow; partial using a grey arrow.

To explore our hypothesis that CRISPR1-CRISPR2 loci and CRISPR3-CRISPR4 loci are functionally linked, we examined their leader sequences. In *E. coli* K12 CRISPR1 is constitutively and unidirectionally transcribed from a promoter within the leader sequence as a long precursor that is further processed into crRNAs [20]. For this reason, we have analyzed how this particular sequence was conserved among CRISPR. Such sequences were only identified in *Escherichia*/*Salmonella* genomes containing CRISPR1. They were found at the expected genomic position (i.e. in the direct vicinity of the first repeat). Leader sequences remain in genomes with degraded CRISPR/*cas* systems (Figure 4). It is the first time that a leader sequence conserved among two distinct genera is observed (>70% identity sequence). In *Salmonella* the leader of CRISPR1 and CRISPR2 are somewhat similar (>65% identity). In all other cases, the two leader sequences, although AT rich, are very different. This result allowed us to orient the CRISPR2 relative to the sense of transcription, and to notice that it has the same transcriptional orientation as the CRISPR1. This matches the expected transcription orientation given the sequenced catalog of crRNAs from *E. coli* [30]. We speculated that the similarity in leader sequences of CRISPR1 and CRISPR2 might result from a recent duplication of CRISPR1 in these chromosomes. However, sequences are very divergent for a recent duplication, the loci are not contiguous, as would be expected for recent amplifications [31], and, as we shall describe below, these two loci do not share any common spacer. One might be tempted to suggest that Cas proteins need specific recognition sequences resulting in high conservation of the leaders. Yet, not only leaders are not highly conserved but divergence is very high between the leader sequences of the putatively active CRISPR1 and CRISPR2 in all *Escherichia* and some *Salmonella* genomes. It's therefore unclear at this stage if the leader has any role in linking CRISPR with the cas system in trans in the genome.

### Spacers turnover is slow and incongruent with cas phylogeny

Spacers flank consecutive repeats and constitute the most diverse part of CRISPR. We found a total of 926 spacers with a characteristic size of ∼32 bp. We noted few exceptions: (i) In *E. coli* ATCC 8739 the CRISPR2 array is disrupted by an IS element. This could provide *cis* regulatory signals enhancing the transcription of distal CRISPR spacers in this especially large repeat-spacer array (32 repeats; Figure 4). (ii) In *S. enterica* CT18 and TY2 the CRISPR1 contains respectively 3 and 2 adjacent overlapping repeats which likely reflect recent loss of spacers. (iii) In 18 *E. coli* the CRISPR2 contains a large (∼0.5 kb) non-coding sequence highly conserved which led [23] to consider the existence of two CRISPR at this position. To investigate the evolutionary dynamics of spacers, we compared them between loci or within the same locus in different genomes. We found that very closely related genomes, at distances lower than 0.02% substitutions per position (Figure 1B), have identical spacers, apart recent integrations of transposable elements. The few differences observed between the genomes CT18 and TY2 and also between AKU_12601 and ATCC9150 are likely to correspond to recent deletions (Figure 4).

We made a coarse estimation of the divergence time of these closely related strains. For this we used the previously published estimate of 7.6×10^−10^ substitutions per year in *E. coli* [32]. Using this rate a 0.02% divergence leads to an estimated divergence time of 250 000 years. Since such molecular clock estimates are subject to some uncertainty we also made an extremely conservative dating by assuming that all mutations accumulate neutrally in genomes, i.e. in the absence of purifying selection. *Escherichia* and *Salmonella* have genomic mutation rates of ∼10^−3^/generation, ∼5 Mb genomes, estimated average doubling times of 40 h in nature [33], and an input of polymorphisms by recombination 2.5 times higher than that of mutation [26]. Under these conditions the 0.02% divergence in neutral loci corresponds to ∼300 000 generations, i.e. over 1 300 years. Note that this estimate is extremely conservative because comparisons between distant *E. coli* strains show an excess of synonymous over non-synonymous rates of a factor of ∼20 [34]. Since synonymous positions are also under selection for codon usage bias this demonstrates strong purifying selection in non-synonymous positions. Both the most accurate and the most conservative estimates show that strains having diverged in the last thousand years have identical CRISPR. This means that CRISPR evolve at an exceedingly slow pace for a putative immune system.

**Figure 5:**
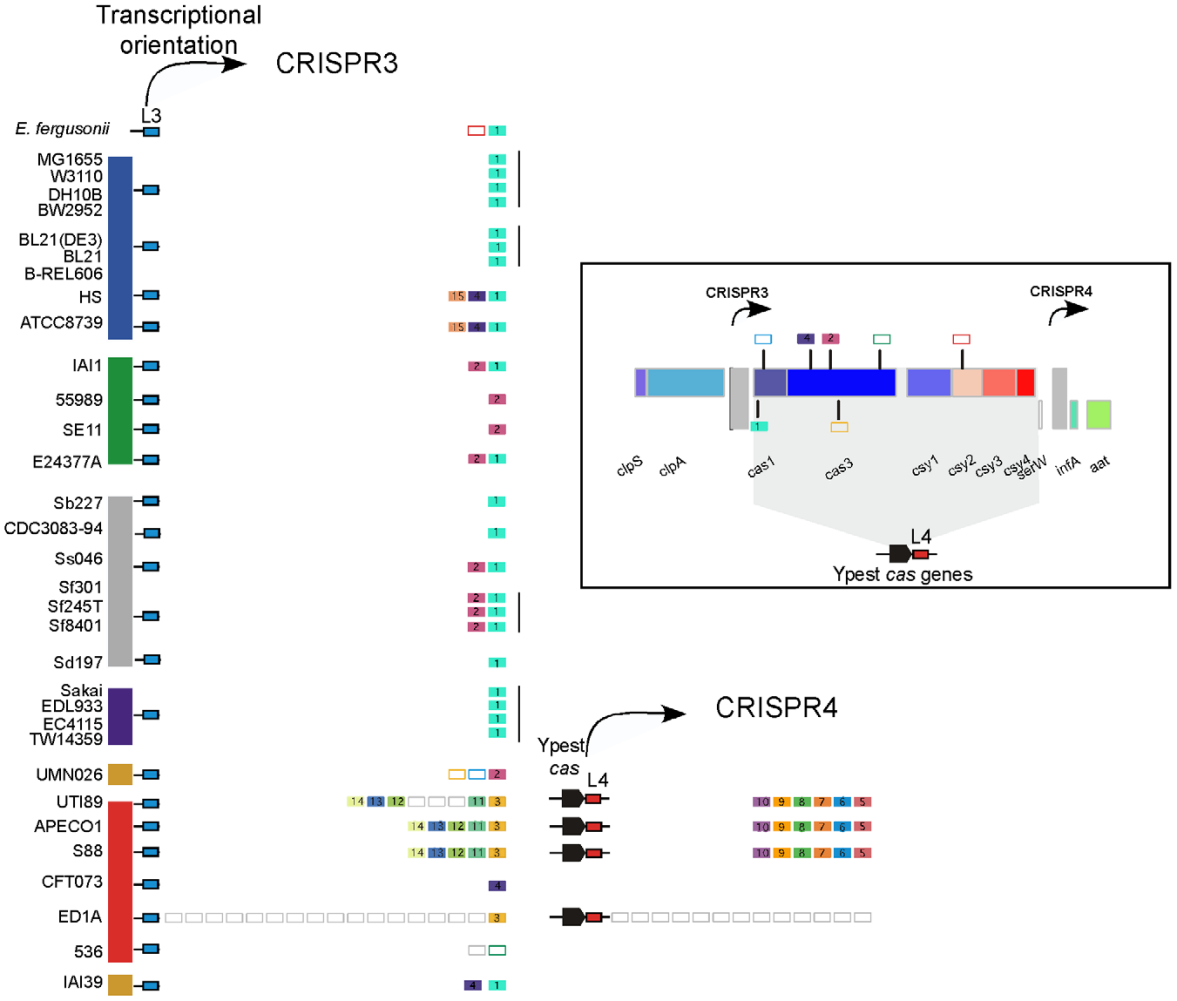
Graphic representation of spacers across CRISPR3 and CRISPR4. Repeats are not included. Single spacers appear in white background; identical spacers are represented using a similar color background scheme and identical number. Strains phylogenetic groups are indicated with colors on the left part of the figure. Very closely related genomes (<0.02% substitutions per position) are indicated by a vertical black line. Similar leaders (called L3 and L4) are represented using identical colour background scheme (i.e. red and blue). Complete Ypest CRISPR/*cas* genes subtype is represented using a black arrow. Insert in right: CRISPR3 spacers matches superimposed on Ypest *cas* genes operon. They are indicated by lines above and below the genomes for the two DNA strands.

To avoid redundancy, we then analyzed the spacers of only one genome per group of closely related strains (genomes marked by black circles were removed, Figure 1B). After this filter, we found that 85% of different spacers are present in only one genome (Figure 4 and Figure 5). These singletons are always located at the 5′ end of the CRISPR (according to transcription orientation). This supports the polarized acquisition of new spacers, with new units being added at one end of the cluster near the leader sequence. On the other end of the CRISPR we find the most conserved spacers. These are likely to be ancient and some are found in nearly all CRISPR of the species. Pairs of conserved spacers are in the same order even in the most distant genomes in the species, despite the low observed rate of duplications and the very high rate of deletions observed in *E. coli* genomes [26]. One is tempted to speculate that either these spacers are intrinsically more stable than the others for some unknown reason, or that they are more strongly selected for and thus rarely lost. The detailed analysis of these conserved spacers reveals some intriguing cases. For example, the CRISPR2 spacer marked 1 in Figure 4 is present in most *E. coli* genomes, including genomes of the group E, B1, *Shigella* and A. This is surprising because the E, B1 and *Shigella* groups have *cas* genes with a different phylogenetic history from the group A (Figure 3). The spacer marked 3 in CRISPR1 also exists in both group E and group A. While several strains of the same species share spacers, there is no spacer common to *E. coli* and *S. enterica*, even though the *cas* phylogeny places groups of *E. coli* and *S. enterica* close together (Figure 3). This strongly suggests that spacers can follow evolutionary paths very different from the ones of the contiguous and functionally linked *cas* genes.

### Proto-spacers suggest CRISPR specialization

Spacers of CRISPR are thought to derive from sub-sequences of mobile genetic elements, named proto-spacers. To unravel the ecological role of CRISPR we searched for proto-spacers of the CRISPR in the available 1725 complete genomes of plasmids, 522 genomes of phages, and 1122 bacterial chromosomes. It was previously shown that perfect identity between proto-spacer and spacer is required to provide immunity [4], [5]. Yet, considering that phage sequences evolve rapidly and that sampling of the viral world is still very incomplete we searched for proto-spacers with a less stringent identity criterion (i.e. >95%). This should allow us to identify the type of the putative target, but not the exact host of the proto-spacer. Despite the profuse availability of phage and plasmid sequences for enterobacteria we only matched proto-spacers for 49 distinct spacers, among 594 (Table 2; Materials and Methods). Spacers were derived both from genic and intergenic regions (Table 2). The high percentage of genic spacers likely reflects the coding density of the genomes (∼87%). As shown previously, we confirmed there is no significant bias to either sense or antisense strands of genes: both strands are targeted to an equal degree (Table 2) [4], [21]. These findings strongly suggest that CRISPR spacers are acquired randomly, and non-directionally, from the virus or plasmid DNA, instead of being generated by reverse transcriptase from virus/plasmid transcripts. The results are also consistent with the hypothesis that the CRISPR spacer transcripts target the virus/plasmid by hybridizing directly to their DNA. That only 8% of all spacers match a known sequence presumably reflects the low levels of sampling of phage-sequence space, and is in agreement with recent estimates of huge untapped phage environmental diversity [2]. On the other hand, we could not find one single exact or approximate match to spacers in the vast majority of sequenced enterophages. This suggests that this large set of spacers is very far from representing the diversity of enterophage targets. Presumably this means that in spite of CRISPR, these strains remain vulnerable to the vast majority of phages.

**Table 2:** Characteristics of the spacer arrays.

	CRISPR1	CRISPR2	CRISPR3	CRISPR4	Total
Number of spacers	386	430	79	31	926
Number of distinct spacers	265	277	33	19	594
Number of single spacers (a)	224 (84%)	235(85%)	33(73%)	13(68%)	505 (85%)
Number of distinct spacers having proto-spacer (b)	18(7%)	7(2%)	17(51%)	7(37%)	49(8%)
Number of proto-spacers	48	20	202	68	338
Number of genic proto-spacers	44	19	172	60	295(87%)
Proto-spacers on the coding strand	5(11%)	15(79%)	105(61%)	26(43%)	151(51%)

(a) very closely related strains were removed of this analysis (d<0.02% substitutions per position).

(b) proto-spacers located inside CRISPR were removed of this analysis.

We classed the proto-spacers in three categories: chromosomal, phage, and plasmid (Table 3). Overall among the 49 matched spacers, 14% showed similarity to viral sequences, 42% to plasmids, and 53% to chromosomes. All phages targeted by spacers are dsDNA bacteriophages known to infect *Escherichia*/*Salmonella* cells. Nearly all of the plasmids recognized by spacers are conjugative or mobilizable in *Escherichia*/*Salmonella* cells. CRISPR1 and CRISPR2 match a small number of proto-spacers in known phages and plasmids (Table S4), but all their chromosomal proto-spacers were localized in prophages. These reflect the targeting of phages by CRISPR. Intriguingly, the spacers of CRISPR3 and CRISPR4 match a very large number of known mobile genetic elements of plasmid origin. Interestingly, they also match the chromosomes, but in this case the proto-spacers are the *cas* genes themselves. This case is described more in detail below.

**Table 3:** Characteristics of the proto-spacers matching protein coding genes^a^.

Location	Protein description
CRISPR1 and CRISPR2	
Chromosome (100% prophage) 47 proto-spacers	DNA cytosine methylase
	Baseplate assembly protein J
	Baseplate assembly protein W
	Major tail sheath protein
	Tail fiber family protein
	Phage major capsid protein E
	DnaG primase-like protein
	Phage integrase family protein
	Phage protein
	Hypothetical protein (extrachromosomal origin)
Phage 8 proto-spacers	Defense against restriction protein
	Putative DNA methyltranserase
	Putative transcriptional activator
	Integrase protein
	Hypothetical protein
Plasmid 8 proto-spacers	Putative DNA modification methylase
	Hypothetical protein
CRISPR3 and CRISPR4	
Chromosome (100% *cas* genes) 28 proto-spacers	Cas1 protein (Ypest subtype)
	Cas3 protein (Ypest subtype)
	Csy2 protein (Ypest subtype)
Plasmid 204 proto-spacers	Putative antirestriction protein
	Putative DNA methyltranserase
	Replication protein RepA
	Conjugal transfert protein
	Hypothetical protein

(a) The number of proteins and proto-spacers is not identical because some proto-spacers match the same protein family and vice-versa. Also, we did not multiply generic descriptions such as Hypothetical protein.

**Figure 6:**
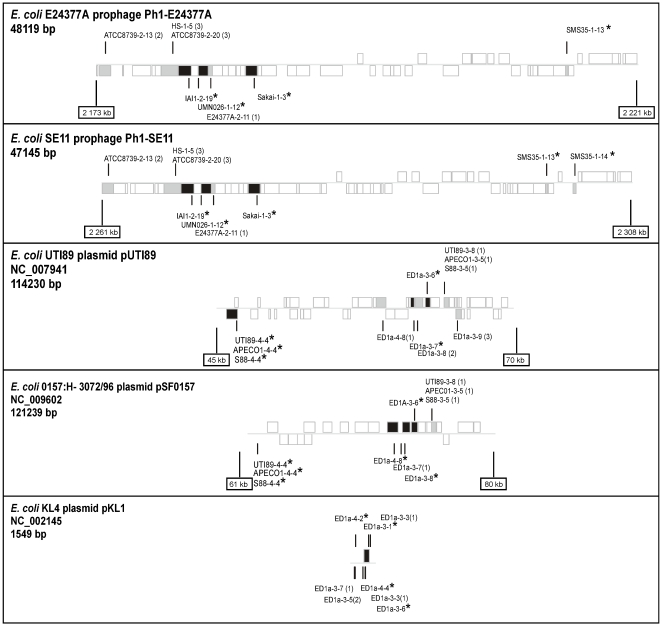
Matches of CRISPR spacers in five different genetic elements including 2 prophages (up), and 3 plasmids (bottom). Protein-coding regions are boxed and shaded, according to their level of similarity with the spacer (perfect identity: black; >95% identity: grey). The positions where CRISPR spacers match the mobile element are indicated by vertical lines above and below the genomes for the two DNA strands. For the two large plasmids we only represent the region in the plasmid where all matches are concentrated. The positions of the prophages on the bacterial chromosomes, and those of the represented regions on the plasmids are also indicated at the bottom of each representation. The nomenclature used for spacers is the following: Name of the strain-CRISPR locus-Position of the spacer along the CRISPR-(Number of mismatches between the spacer and the proto-spacer). A perfect match is indicated with a star.

Some CRISPR spacers match their own genome prophages or plasmids. Indeed, in *E. coli* strain E24377A one spacer matches a proto-spacer in its own prophage (Figure 6). In *E. coli* strains UTI89, APECO1 and S88, two spacers of CRISPR3 and CRISPR4 respectively targeting sequences of their own plasmids (Figure 6). It is hard to reconcile these results with chromosomal integrity if CRISPR target genetic elements for degradation. Hence, these observations suggest a microbial regulatory role of CRISPR, perhaps using a system based on interference [7], [13].

We then analyzed the genetic context of proto-spacers and found that CRISPR can have from 1 to 8 spacers targeting proto-spacers in the same genome. This suggests multiple acquisitions of spacers in a single infection (see ED1a Figure 6). The order of spacers within a CRISPR did not correspond to the order of matching regions along the virus/plasmid genome. The prevalence of a single repeat-spacer unit in most CRISPR might thus simply reflect spacer loss due to absence of selection for multiple spacers matching the same mobile element. Recent analyses have shown proto-spacers uniformly distributed throughout the virus/plasmid genomes [35]. In fact, if we consider separately each CRISPR their proto-spacers seem to be clustered in small regions. For example, 5 proto-spacers targeted by the CRISPR3 and CRISPR4 of *E. coli* strain ED1a are clustered in a small region of 3 kb whereas the plasmid pSFO157 is 121 kb long (Figure 6). This suggests that within a genome the spacers of the CRISPR derived from a limited region of the virus/plasmid. The joint presence of spacers from the same plasmid in CRISPR3 and CRISPR4 strongly supports our hypothesis that these two CRISPR are functionally coupled. Interestingly, 10 spacers of the CRISPR3 and CRISPR4 of *E.coli* strain ED1a showed perfect matches with 35 distinct plasmids (see insert Figure 6). This number rises to 57 plasmids when using a less stringent identity criterion (i.e. >95%). Thus, these CRISPR seem to provide wide-range protection against plasmids, which could explain why this strain is one of the few strains belonging to the group B2 not to be pathogenic [26], plasmids often carrying virulence-related genes [26].

We have also observed that different spacers from CRISPR of different strains can target the same virus/plasmid/prophage. Indeed, as also found by [23], distinct matches with 10 *E. coli* strains were found across two closely related *E. coli* prophage genomes, named Ph1-E24377A and Ph1-SE11 (Figure 6). Thus, most *E. coli* cells belonging to A, B1, D and E groups could be resistant to this class of prophages. This suggests that these cells have become resistant to the same phage through convergent evolution involving distinct acquisition or selection of particular spacers. This is consistent with our previous suggestion of random spacers acquisition from short virus/plasmid regions. It also suggests a recent global epidemic of this class of phages.

### CRISPR, restriction, anti-restriction and anti-CRISPR

Several different tools are used in the arms races between prokaryotes, phages and plasmids. One of them is the use of restriction and modification systems (RMS). RMS can be used by prokaryotes to avoid phages and plasmids, by plasmids to compete with other plasmids and by plasmids to avoid segregation from the cell [36]. As a response, many phages and plasmids have developed strategies to avoid degradation of their DNA by these systems. Such mechanisms, called anti-restriction systems, involve modification of the phage genome (methylase proteins), transient occlusion of restriction sites, subversion of host RMS activities (activation of host methylation), and direct inhibition of restriction enzymes (antirestriction proteins) [3]. Interestingly, our analysis suggests that CRISPR often target RMS both in phages and in plasmids (Table 3). Hence, CRISPR might complement the action of RMS by barring mobile genetic elements able to sideline RMS.

CRISPR systems have often been acquired through horizontal gene transfer mediated by plasmids or phages. But, why should CRISPR exist in phages and plasmids? CRISPR might mediate antagonism between genetic elements; just like Toxin-Antitoxin and restriction and modification systems are used for competition between plasmids. The acquisition of a mobile element with a CRISPR might pose a serious danger to the host if the CRISPR contains spacers matching the chromosome. The outcome could be chromosome degradation if CRISPR/*cas* systems target DNA degradation. If CRISPR/*cas* systems only lead to gene expression interference, this could still allow host manipulation by the mobile element. Genomes might thus have evolved mechanisms to escape or inhibit incoming CRISPR/*cas* systems. This could include the use of native CRISPR acting as anti-CRISPR, i.e. as a set of spacers that upon functioning of the CRISPR system, e.g. by action of a cognate *cas* system in a mobile element, leads to inactivation of the matched *cas* genes. The CRISPR3 present in all *E. coli* except those belonging to the group B2 could be an anti-CRISPR. Indeed, the spacers marked 1 and 2 (Figure 5) showed identity to the Cas1 and Cas3 proteins belonging to the Ypest CRISPR/*cas* subtype. Overall, 7 distinct spacers in CRISPR3 have similarity with Cas1, Cas3 and Csy2 Ypest proteins (Figure 5). Interestingly, at least one of these spacers is present in all the CRISPR3 of *E. coli* genomes lacking Ypest *cas* genes. The correlation between the presence of these particular spacers and the specific lack of Ypest *cas* genes subtype strongly suggest that this residual CRISPR could be a functional anti-CRISPR Ypest system. If so, the ancestor of B2 might have lost the spacer, rendering it receptive to the acquisition of the *cas* system, leading to spacers turnover and to the creation of CRISPR4. CRISPR3 of genomes lacking Ypest *cas* system contain only spacers matching *cas* genes of its specific subtype system. Anti-CRISPR are very short which is expected if acquisition of new spacers is generally prevented. Hence, these results are concordant with our hypothesis that residual CRISPR spacers targeting *cas* genes could prevent invasion by genetic elements containing functional CRISPR.

### Conclusion

*E. coli* CRISPR have been identified before and the CRISPR1 has been well-described [12], [23], [24], [37], [38]. We confirmed several previous observations such that spacers are taken up randomly and non-directionally. We have however observed putative multiple acquisitions of spacers. Interestingly, the presence of stretches of conserved repeat variants strongly suggests that the new repeat sequence comes from the duplication of the adjacent repeat sequence (e.g. Table S6). When present in genomes, CRISPR are always located at the same locations despite the multiple occurrences of *cas* genes degradation and *cas* horizontal transfer. This implies that the process of replenishing genomes with intact *cas* loci is frequent and that horizontally transferred *cas* genes are always inserted in the same locations, next to a given CRISPR. We propose that CRISPR might out-live the *cas* genes in the genome, thereby providing for an integration hotspot. This is most clearly demonstrated by the observation that sub-clades with different *cas* genes contain some similar spacers. We have shown that CRISPR1 and CRISPR2 on one hand and CRISPR3 and CRISPR4 on the other are functionally coupled: these pairs are co-localized in the genome, they have identical repeats, they are associated with similar CRISPR/*cas* genes subtypes, and tend to show correlated dynamics. These results are in agreement with previous suggestions [11], [23] and with recent experimental data showing co-regulation of CRISPR1 and CRISPR2 by the global pleiotropic regulator H-NS [30]. CRISPR/*cas* subtypes might therefore be specialized and act in trans on all CRISPR with identical repeat sequences. Interestingly, the analysis of the spacers strongly suggests that CRISPR1 and CRISPR2 target mostly phages, whereas CRISPR3 and CRISPR4 only target plasmids. Previous works have shown that genomes containing multiple CRISPR rarely exhibit more than one or two loci reactive to infection [6]. It is tempting to speculate that this might also be due to CRISPR specialization in those genomes. Why and how CRISPR are specialized remains unknown but one could imagine different mechanisms aiming at responding to incoming DNA, e.g. one mechanism for dsDNA phages, one for ssDNA phages and incoming conjugative plasmids and one for RNA phages.

This study supports the idea that new spacers are acquired in a polarized fashion, with new units being added at the leader end of the CRISPR. This implies that spacers are chronological records reflecting previous encounters with mobile genetic elements [4]. However, the loss of one or more repeat-spacer units has been observed. This suggests that CRISPR do not grow unchecked. One would assume that older spacers should be more frequently deleted because they have been inserted for a longer time. Not only older spacers had longer opportunity for deletion but they also match ancient, instead of extant, mobile elements. Surprisingly, some of the most ancient spacers are highly persistent and thus shared by nearly all CRISPR of the species. This might indicate a critical unknown function in CRISPR/*cas* system activity. Our results also suggest that periods of *cas*-activity in the genome are associated with increase in CRISPR arrays and that the remaining periods are associated with the loss of spacers. Hence, while closely related strains have essentially identical CRISPR, more distantly related strains have radically different CRISPR. Considering that all these genomes contain relatively few spacers the relevance of using CRISPR for typing and epidemiological studies is questionable in enterobacteria, even if it has been shown valuable in other clades such as *Mycobacterium* and *Campylobacter* [39], [40].

CRISPR are consistently described as among the most rapidly evolving genomic loci because of their presumed evolutionary role as an immunity system. In the present case and considering the high genetic variability of *E. coli* genomes [26], the CRISPR seem remarkably static. None of the *Escherichia/Salmonella* genomes analyzed in this work has more than 3 CRISPR, whereas *Methanocaldococcus jannaschii* has 18. The positions of the CRISPR are strictly conserved and no locus has more than 34 repeat-spacers units, whereas the thermophilic bacterium *Chloroflexus* sp. Y-400-fl has 375. In addition, strains that have diverged in the last 250 000 years show no single insertion of new spacers showing a remarkably slow turnover relative to the species generation time and to what one would expect from the known dynamics of bacteria-phage interactions [41]. Despite, the outstanding opportunity provided by the availability of many sequenced enterophages, unlike in other clades, only 7% of these elements were matched by CRISPR spacers, and this while tolerating more mismatches than the CRISPR system seems to tolerate. Presumably this means that in spite of CRISPR, these strains remain vulnerable to the vast majority of phages. Accordingly, the susceptibility of 59 coliphages was not found to correlate with the size of CRISPR, and the *E. coli* cells that effectively survived these phages did not show changes in CRISPR [23]. One could imagine that strains are only resistant to phages encountered locally, and that these were highly specific. However, the available data suggests that phages disperse very fast and are present in many different environments [2]. These results seriously raise the question of CRISPR efficiency in providing wide-range protection against phages in enterobacteria. Since CRISPR are under purifying selection it is tempting to speculate that they might also perform other cellular functions.

Our results are consistent with previous reports on the high transmissibility of CRISPR and their association with plasmids, megaplasmids and even prophages. Why CRISPR exist in phages and plasmids remains unknown. Mobile elements with CRISPR containing spacers matching the host chromosome could have highly deleterious effects. We are inclined to believe that residual CRISPR may, under certain circumstances, confer selective advantages to their host cells and, in these cases, stabilize the loci against degradation. This suggestion is strongly supported by the finding of one short CRISPR containing only spacers matching *cas* genes of its own subtype in all genomes devoid of the corresponding *cas* genes. One might suppose that acquisition of these spacers led to selection of the loss of *cas* genes as proposed very recently by Diez-Villasenor et al [23]. Yet, there are three strong arguments against this hypothesis. First, if functional *cas* are selected for and if anti-CRISPR perform no effective role, then selection should have removed the variants with anti-CRISPR and lacking *cas* not the other way around. Instead, the variants with anti-CRISPR and without *cas* are far more abundant. Note that simple evolutionary inertia cannot explain the persistence of anti-CRISPR in the lineages because spacers are expected to be frequently deleted by illegitimate recombination in *E. coli* [42]. This is indeed observed for most other spacers, but not for the ones matching *cas* genes, strongly suggesting that these spacers are selected for. Second, the phylogenetic analyses depicted in Figures 3 and 5 and the observed conservation of the terminal repeat sequence in all *E. coli* genomes lacking Ypest *cas* genes (Table S5) show that these anti-CRISPR spacers pre-date the extant *cas* genes. Third, *cas*-less CRISPR3 are found in most branches of *E. coli*, whereas *cas*-containing CRISPR3 are monophyletic. As a result, a loss of *cas* genes requires multiple parallel events of loss whereas the *cas* acquisition only requires one transfer. Our hypothesis is therefore more parsimonious. We have shown here by phylogenetic analysis that *cas* genes are indeed frequently transferred. Along this line, the most likely scenario is the transfer of a *cas*-less anti-CRISPR into *Escherichia* followed by the acquisition of the *cas* system in the B2 group after loss of the anti-CRISPR. We therefore propose that CRISPR themselves can be used to prevent the invasion of mobile elements carrying functional CRISPR/*cas* systems. The study of CRISPR is in its infancy, and their functioning and role subject to considerable uncertainty. Our results provide an example of how evolutionary works using full closely related genome data might contribute to a comprehensive understanding of these intriguing elements.

## Materials and Methods

### Data

We analyzed 51 complete genomes of *Escherichia* and *Salmonella* species, taken from GenBank genomes (ftp://ftp.ncbi.nih.gov/genomes/). These include 27 strains of *E. coli*, 7 strains of *Shigella* (which in fact are strains from *E. coli*), 16 strains of *Salmonella* and 1 strain of *E. fergusonii*. We also analysed 1725 publicly available plasmid genomes, 522 phage genomes and 1122 bacterial genomes. We used GenBank annotations, excluded genes with stops in phase and with lengths not multiple of three. We also re-annotated the prophage and the transposases using respectively Phage-Finder [43] and a program developed in our laboratory [44] (Touchon, unpublished).

### Assignment of orthology

A preliminary set of orthologs was defined by identifying unique pairwise reciprocal best hits, with at least 60% similarity in amino acid sequence and less than 20% of difference in protein length. Because few rearrangements are observed at these short evolutionary distances, genes outside conserved blocks of synteny are likely to be xenologs or paralogs. Hence, this list was then refined by combining the information on the distribution of similarity of these putative orthologs and the data on gene order conservation. The analysis of orthology was made for every pair of *Escherichia/Salmonella* genomes. The core genome consists of genes found in all strains of the species and was defined as the intersection of pairwise lists.

### Phylogenetic analyses

The reference phylogenetic tree for the core genome was reconstructed from the concatenated alignments of 1241 proteins of the core genome obtained with muscle v3.6 [45] then back-translated to DNA, as is standard usage. We used Tree-puzzle 5.2 [46], to compute the distance matrix between all genomes using maximum likelihood under the HKY + G(8) + I model. The tree of the core genome was built from the distance matrix using BioNJ [47]. We made 1000 bootstrap experiments on the concatenated sequences to assess the robustness of the topology. The topology of this tree is congruent with previous whole-genome phylogenetic analyses of *E. coli* (e.g. [26]).

The molecular phylogeny of all Cas proteins has been explored by the construction of multiple sequence alignments with muscle v3.6 [45]. After alignment, ambiguous regions (i.e. containing gaps and/or poorly aligned) were removed with Gblocks (v0.91b) [48]. The phylogenetic tree was reconstructed using the maximum likelihood method implemented in the PhyML program (v3.0 aLRT) with the WAG matrix and a gamma correction for variable evolutionary rates [49]. Reliability for internal branch was assessed using the aLRT test [50].

### CRISPR identification

CRISPR were identified using CRT (CRISPR Recognition Tool) with default parameter values [51], in the 51 *Escherichia/Salmonella* complete genomes, then in the 1122 bacterial and archaeal genomes, 522 phage genomes, and 1725 plasmid genomes. In the 51 complete genomes, loci bordered by the same core genes were identified as CRISPR1 (bounded by the 2 core genes: *cysD-cysJ*), CRISPR2 (*cysJ-ygcF*), CRISPR3 (*clpS-tRNA_Ser_*), *and* CRISPR4 (*tRNA_Ser_-infA*) (Figure 1A). For each CRISPR, the repeats were extracted and were aligned using Muscle [45]. Then we used Cons (http://bioweb.pasteur.fr/docs/EMBOSS/cons.html) to obtain consensus sequences from these 4 multiple sequence alignments. In all these cases, the consensus sequence corresponds to the most frequent sequence within a particular CRISPR.

We used the repeats patterns to identify additional, smaller and/or degenerate repeat clusters in the 4 CRISPR-associated genomic regions (i.e. *cysD-cysJ*; *cysJ-ygcF*; *clpS-tRNA_Ser_* and *tRNA_Ser_-infA*) in the 51 *Escherichia/Salmonella* complete genomes with Fuzznuc (http://bioweb2.pasteur.fr/docs/EMBOSS/fuzznuc.html). Thus, CRISPR identified in this work may have one single repeat, they may be degenerate (at least 60% of identity but with identical sequence length), and they may have irregularly spaced repeats due to a transposase insertion or due to the lack of one spacer. Once we identified the repeats we extracted the spacers of each locus.

The alignments of the repeats of each CRISPR pairs were visualized with WebLogo version 3.0 (http://weblogo.threeplusone.com/create.cgi), a Web-based application that generates graphical representations (logos) of the patterns within a multiple sequence alignment [52]. Gaps were not added in any case. Each logo consists of stacks of letters, one stack for each position in the sequence. The height of letters within the stack reflects the relative frequency of the corresponding nucleotide at that position.

Secondary structural prediction of the most frequent sequence within each CRISPR pairs were performed using Mfold (http://mfold.bioinfo.rpi.edu/cgi-bin/rna-form1.cgi) [53].

### cas gene identification

The Hidden Markov models (HMMs) for the 45 Cas protein families described in [12] were obtained from the TIGRFAM database, version 6.0 (http://www.tigr.org/TIGRFAMs/). To identify *cas* genes, all coding sequences within the 4 CRISPR-associated genomic regions were searched with the Cas HMMs profiles using hmmpfam [54] with the thresholds of an e-value <0.001 and a positive score. To identify *cas* pseudogene, all Cas proteins previously detected were searched in all the genomic sequences analysed using tbastn. This step also allowed to check the absence of these *cas* genes in other locations along the *Escherichia*/*Salmonella* chromosomes and plasmids. A similar method was used to check the presence of complete Ypest *cas* genes subtype in phylogenetically distant chromosomes and plasmids.

### Leader sequence identification

To identify leader, the well-known CRISPR1 leader sequence of *E. coli* K12 was searched in all the genomic sequences analyzed using blastn. Thus we identified the leader in most CRISPR1 of *Escherichia* and *Salmonella*, and in all CRISPR2 of *Salmonella* (see yellow box in Figure 4). In all other cases, leaders were identified as large conserved sequences adjacent to each CRISPR locus. Dissimilar CRISPR surroundings permit a more confident identification of the leader as a conserved sequence at just one side of the array. Significance was evaluated by comparison with alignments of the opposite CRISPR flanking region. The presence in the putative leader of A or T tracks, and the occurrence of a degenerated repeat in the distal end of the CRISPR were confirmed [8].

## Supporting Information

Table S1 CRISPR array flanked by Ecoli *cas* genes subtype. Occurrences of the 8 Ecoli *cas* genes subtype per genome. 0 = no gene present, 1 = gene present, P = pseudogene present in the genome. Genome Sequences. The strain name referenced throughout the manuscript. Accession number. NCBI Accession number. Phylogenetic Group. See Figure 1.
(0.15 MB DOC)

Table S2 CRISPR array flanked by Ypest *cas* genes subtype. Occurrences of the 6 Ypest *cas* genes subtype per genome. 0 = no gene present, 1 = gene present, P = pseudogene present in the genome. Genome Sequences. The strain name referenced throughout the manuscript. Accession number. NCBI Accession number. Phylogenetic Group. See Figure 1.
(0.13 MB DOC)

Table S3 The complete Ypest *cas* system is present in phylogenetically distant chromosomes and plasmids.
(0.05 MB DOC)

Table S4 CRISPR1 and CRISPR2 match a small number of proto-spacer in the following known phages and plasmids.
(0.03 MB DOC)

Table S5 Conservation of the terminal repeat sequence of the CRISPR3 and CRISPR4.
(0.08 MB DOC)

Table S6 Example of stretches of conserved repeat variants among the CRISPR array.
(0.04 MB DOC)
