# Supplementary material for: The Small, Slow and Specialized CRISPR and Anti-CRISPR of Escherichia and Salmonella
Source: PLoS One. 2010 Jun 15;5(6):e11126. doi: 10.1371/journal.pone.0011126 (PMC2886076; doi:10.1371/journal.pone.0011126)
Supplement: Table S1 — CRISPR array flanked by Ecoli cas genes subtype. Occurrences of the 8 Ecoli cas genes subtype per genome. 0 = no gene present, 1 = gene present, P = pseudogene present in the genome. Genome Sequences. The strain name referenced throughout the manuscript. Accession number. NCBI Accession number. Phylogenetic Group. See Figure 1. (0.15 MB DOC) [file pone.0011126.s001.doc]

| ***Genome Sequences*** | ***Accession Number*** | ***Phylogenetic-Group*** | ***Core-genomic-Begin cysD*** | ***Core-Genomic-End ygcF*** | ***Locus-Size CRISPR1+cas***  ***+CRISPR2*** | ***Cas2*** | ***Cas1*** | ***Cse3*** | ***Cas5*** | ***Cse4*** | ***Cse2*** | ***Cse1*** | ***Cas3*** | ***N_repeat CRISPR1*** | ***N_repeat CRISPR2*** |
| --- | --- | --- | --- | --- | --- | --- | --- | --- | --- | --- | --- | --- | --- | --- | --- |
| *Salmonella enterica arizonae* SL62 | NC_010067.1 | Sa | 16973 | 27781 | 10808 | 0 | 0 | 0 | 0 | 0 | 0 | 0 | P | 2 | 0 |
| *Salmonella enterica serovar Schwarzengrund* CVM19633 | NC_011094.1 | Sa | 2979729 | 3001502 | 21773 | 1 | 1 | 1 | 1 | 1 | 1 | 1 | 1 | 13 | 18 |
| *Salmonella enterica Typhi* CT18 | NC_003198.1 | Sa | 2923962 | 2944180 | 20218 | 1 | 1 | 1 | 1 | 1 | 1 | 1 | P | 8 | 2 |
| Salmonella enterica Typhi TY2 | NC_004631.1 | Sa | 2909821 | 2930074 | 20253 | 1 | 1 | 1 | 1 | 1 | 1 | 1 | P | 8 | 2 |
| *Salmonella enterica serovar Paratyphi A* AKU_12601 | NC_011147.1 | Sa | 2882885 | 2903290 | 20405 | 1 | 1 | 1 | 1 | 1 | 1 | P | P | 8 | 4 |
| *Salmonella enterica serovar Paratyphi A* ATCC9150 | NC_006511.1 | Sa | 2887349 | 2907632 | 20283 | 1 | 1 | 1 | 1 | 1 | 1 | P | P | 6 | 4 |
| *Salmonella typhimurium* LT2 | NC_003197.1 | Sa | 3074391 | 3097228 | 22837 | 1 | 1 | 1 | 1 | 1 | 1 | 1 | 1 | 26 | 33 |
| *Salmonella enterica serovar Heidelberg* SL476 | NC_011083.1 | Sa | 3048997 | 3071231 | 22234 | 1 | 1 | 1 | 1 | 1 | 1 | 1 | 1 | 28 | 19 |
| *Salmonella enterica serovar Cholerasuis* SC-B67 | NC_006905.1 | Sa | 3029313 | 3050666 | 21353 | 1 | 1 | 1 | 1 | 1 | 1 | P | P | 5 | 8 |
| *Salmonella enterica serovar Paratyphi C* RKS4594 | NC_012125.1 | Sa | 3008384 | 3030226 | 21842 | 1 | 1 | 1 | 1 | 1 | 1 | 1 | 1 | 11 | 10 |
| *Salmonella enterica serovar Paratyphi B* SPB7 | NC_010102.1 | Sa | 3039109 | 3052105 | 12996 | 0 | 0 | 0 | 0 | 0 | 0 | 0 | P | 3 | 6 |
| *Salmonella enterica serovar Gallinarum* SL287/91 | NC_011274.1 | Sa | 2949954 | 2970085 | 20131 | 1 | 1 | 1 | 1 | 1 | 1 | 1 | P | 3 | 11 |
| *Salmonella enterica serovar Enteritidis* P125109 | NC_011294.1 | Sa | 2959150 | 2979645 | 20495 | 1 | 1 | 1 | 1 | 1 | 1 | 1 | 1 | 9 | 11 |
| *Salmonella enterica serovar Dublin* CT_02021853 | NC_011205.1 | Sa | 3118881 | 3138710 | 19829 | 1 | 1 | 1 | 1 | 1 | 1 | 1 | P | 3 | 6 |
| *Salmonella enterica serovar Newport* SL254 | NC_011080.1 | Sa | 3052639 | 3075296 | 22657 | 1 | 1 | 1 | 1 | 1 | 1 | 1 | 1 | 27 | 20 |
| *Salmonella enterica Agona str.* SL483 | NC_011149.1 | Sa | 2985885 | 3007001 | 21116 | 1 | 1 | 1 | 1 | 1 | 1 | 1 | 1 | 19 | 9 |
| *Escherichia fergusonii* | NC_011740.1 | F | 310183 | 330457 | 20274 | 1 | 1 | 1 | 1 | 1 | 1 | 1 | 1 | 20 | 17 |
| *Escherichia coli* MG1655 | NC_000913.2 | A | 2873443 | 2903440 | 29997 | 1 | 1 | 1 | 1 | 1 | 1 | 1 | 1 | 13 | 10 |
| *Escherichia coli* W3110 | AC_000091.1 | A | 2874077 | 2904074 | 29997 | 1 | 1 | 1 | 1 | 1 | 1 | 1 | 1 | 13 | 10 |
| *Escherichia coli* DH10B | NC_010473.1 | A | 2965985 | 2995982 | 29997 | 1 | 1 | 1 | 1 | 1 | 1 | 1 | 1 | 13 | 10 |
| *Escherichia coli* BW2952 | NC_012759.1 | A | 2759255 | 2789252 | 29997 | 1 | 1 | 1 | 1 | 1 | 1 | 1 | 1 | 13 | 10 |
| *Escherichia coli* BL21(DE3) | NC_012947.1 | A | 1001897 | 1024515 | 22618 | 0 | 0 | 0 | 0 | 0 | 0 | 0 | 0 | 6 | 17 |
| Escherichia coli BL21 | NC_012892.1 | A | 2714599 | 2737216 | 22617 | 0 | 0 | 0 | 0 | 0 | 0 | 0 | 0 | 6 | 17 |
| *Escherichia coli* B-REL606 | NC_012967.1 | A | 2770282 | 2794346 | 24064 | 0 | 0 | 0 | 0 | 0 | 0 | 0 | 0 | 6 | 17 |
| *Escherichia coli* HS | NC_009800.1 | A | 2892840 | 2922850 | 30010 | 1 | 1 | 1 | 1 | 1 | 1 | 1 | 1 | 11 | 20 |
| *Escherichia coli* ATCC8739 | NC_010468.1 | A | 1015289 | 1048463 | 33174 | 1 | 1 | 1 | 1 | 1 | 1 | 1 | 1 | 22 | 32 |
| *Escherichia coli* IAI1 | NC_011741.1 | B1 | 2924432 | 2955795 | 31363 | 1 | 1 | 1 | 1 | 1 | 1 | 1 | 1 | 18 | 25 |
| *Escherichia coli* 55989 | NC_011748.1 | B1 | 3091933 | 3121955 | 30022 | 1 | 1 | P | 1 | 1 | 1 | 1 | 1 | 2 | 19 |
| *Escherichia coli* SE11 | NC_011415.1 | B1 | 3112741 | 3144352 | 31611 | 1 | 1 | 1 | 1 | 1 | 1 | 1 | 1 | 23 | 24 |
| *Escherichia coli* E24377A | NC_009801.1 | B1 | 3054191 | 3085495 | 31304 | 1 | 1 | 1 | 1 | 1 | 1 | 1 | 1 | 13 | 29 |
| *Shigella boydii* Sb227 | NC_007613.1 | Sh | 2650616 | 2777858 | 127242 | 1 | P | 1 | 1 | P | 1 | 1 | P | 1 | 3 |
| *Shigella boydii* CDC3083-94 | NC_010658.1 | Sh | 2897169 | 2916854 | 19685 | 1 | P | 1 | 1 | 0 | 0 | 0 | 0 | 1 | 1 |
| *Shigella sonnei* Ss046 | NC_007384.1 | Sh | 3039065 | 3071661 | 32596 | 1 | P | 0 | 1 | 1 | P | P | 1 | 3 | 4 |
| *Shigella flexneri 2a* Sf301 | NC_004337.1 | Sh | 2847558 | 2864303 | 16745 | 0 | 0 | 0 | 0 | 0 | 0 | 0 | P | 1 | 0 |
| *Shigella flexneri 2a* Sf2457T | NC_004741.1 | Sh | 2842262 | 2859007 | 16745 | 0 | 0 | 0 | 0 | 0 | 0 | 0 | P | 1 | 0 |
| *Shigella flexneri 2a* Sf8401 | NC_008258.1 | Sh | 2754863 | 2816460 | 61597 | 0 | 0 | 0 | 0 | 0 | 0 | 0 | P | 1 | 0 |
| *Shigella dysenteriae* Sd197 | NC_007606.1 | Sh | 2728536 | 2756220 | 27684 | 1 | P | 1 | P | 0 | 0 | 0 | 0 | 5 | 1 |
| *Escherichia coli* O157:H7 Sakai | NC_002695.1 | E | 3595944 | 3625226 | 29282 | 1 | 1 | 1 | 1 | 1 | 1 | 1 | 1 | 6 | 5 |
| *Escherichia coli* O157:H7 EDL933 | NC_002655.2 | E | 3663242 | 3692524 | 29282 | 1 | 1 | 1 | 1 | 1 | 1 | 1 | 1 | 5 | 5 |
| *Escherichia coli* O157:H7 EC4115 | NC_011353.1 | E | 3700341 | 3729623 | 29282 | 1 | 1 | 1 | 1 | 1 | 1 | 1 | 1 | 5 | 5 |
| *Escherichia coli* TW14359 | NC_013008.1 | E | 3655066 | 3684349 | 29283 | 1 | 1 | 1 | 1 | 1 | 1 | 1 | 1 | 5 | 5 |
| *Escherichia coli* UMN026 | NC_011751.1 | D | 3179254 | 3209889 | 30635 | 1 | 1 | 1 | 1 | 1 | 1 | 1 | 1 | 19 | 13 |
| *Escherichia coli* UTI89 | NC_007946.1 | B2 | 3037604 | 3057283 | 19679 | 0 | 0 | 0 | 0 | 0 | 0 | 0 | 0 | 0 | 2 |
| *Escherichia coli* APECO1 | NC_008563.1 | B2 | 3052182 | 3071789 | 19607 | 0 | 0 | 0 | 0 | 0 | 0 | 0 | 0 | 0 | 2 |
| *Escherichia coli* S88 | NC_011742.1 | B2 | 2969494 | 2989172 | 19678 | 0 | 0 | 0 | 0 | 0 | 0 | 0 | 0 | 0 | 2 |
| *Escherichia coli* CFT073 | NC_004431.1 | B2 | 3158631 | 3178308 | 19677 | 0 | 0 | 0 | 0 | 0 | 0 | 0 | 0 | 0 | 2 |
| *Escherichia coli* ED1a | NC_011745.1 | B2 | 3140637 | 3160252 | 19615 | 0 | 0 | 0 | 0 | 0 | 0 | 0 | 0 | 0 | 1 |
| *Escherichia coli* 536 | NC_008253.1 | B2 | 2870321 | 2896253 | 25932 | 0 | 0 | 0 | 0 | 0 | 0 | 0 | 0 | 0 | 0 |
| Escherichia coli O127:H6 E2348/69 | NC_011601.1 | B2 | 3110336 | 3137368 | 27032 | 0 | 0 | 0 | 0 | 0 | 0 | 0 | 0 | 0 | 0 |
| *Escherichia coli* IAI39 | NC_011750.1 | D | 3044553 | 3074070 | 29517 | 0 | 0 | 0 | 0 | 0 | 0 | 0 | 0 | 0 | 0 |
| *Escherichia coli* SMS35 | NC_010498.1 | D | 2948619 | 2984672 | 36053 | 1 | 1 | 1 | 1 | 1 | 1 | 1 | 1 | 22 | 0 |
