# Supplementary material for: The Small, Slow and Specialized CRISPR and Anti-CRISPR of Escherichia and Salmonella
Source: PLoS One. 2010 Jun 15;5(6):e11126. doi: 10.1371/journal.pone.0011126 (PMC2886076; doi:10.1371/journal.pone.0011126)
Supplement: Table S2 — CRISPR array flanked by Ypest cas genes subtype. Occurrences of the 6 Ypest cas genes subtype per genome. 0 = no gene present, 1 = gene present, P = pseudogene present in the genome. Genome Sequences. The strain name referenced throughout the manuscript. Accession number. NCBI Accession number. Phylogenetic Group. See Figure 1. (0.13 MB DOC) [file pone.0011126.s002.doc]

| ***Genome Sequences*** | ***Accession Number*** | ***Phylogenetic-Group*** | ***Core-genomic-Begin clpS*** | ***Core-Genomic-End aat*** | ***Locus-Size CRISPR3+cas***  ***+CRISPR4*** | ***cas1*** | ***cas3*** | ***csy1*** | ***csy2*** | ***csy3*** | ***csy4*** | ***N_repeat CRISPR3*** | ***N_repeat CRISPR4*** |
| --- | --- | --- | --- | --- | --- | --- | --- | --- | --- | --- | --- | --- | --- |
| *Salmonella enterica arizonae* SL62 | NC_010067.1 | Sa | 1945559 | 1952832 | 7273 | 0 | 0 | 0 | 0 | 0 | 0 | 0 | 0 |
| *Salmonella enterica serovar Schwarzengrund*  CVM19633 | NC_011094.1 | Sa | 1032748 | 1040068 | 7320 | 0 | 0 | 0 | 0 | 0 | 0 | 0 | 0 |
| *Salmonella enterica Typhi* CT18 | NC_003198.1 | Sa | 934046 | 942118 | 8072 | 0 | 0 | 0 | 0 | 0 | 0 | 0 | 0 |
| Salmonella enterica Typhi TY2 | NC_004631.1 | Sa | 2038959 | 2047031 | 8072 | 0 | 0 | 0 | 0 | 0 | 0 | 0 | 0 |
| *Salmonella enterica serovar Paratyphi A* AKU_12601 | NC_011147.1 | Sa | 1916137 | 1926506 | 10369 | 0 | 0 | 0 | 0 | 0 | 0 | 0 | 0 |
| *Salmonella enterica serovar Paratyphi A* ATCC9150 | NC_006511.1 | Sa | 1920954 | 1931323 | 10369 | 0 | 0 | 0 | 0 | 0 | 0 | 0 | 0 |
| *Salmonella typhimurium* LT2 | NC_003197.1 | Sa | 1022347 | 1031581 | 9234 | 0 | 0 | 0 | 0 | 0 | 0 | 0 | 0 |
| *Salmonella enterica serovar Heidelberg* SL476 | NC_011083.1 | Sa | 1026900 | 1035945 | 9045 | 0 | 0 | 0 | 0 | 0 | 0 | 0 | 0 |
| *Salmonella enterica serovar Cholerasuis* SC-B67 | NC_006905.1 | Sa | 1007369 | 1015512 | 8143 | 0 | 0 | 0 | 0 | 0 | 0 | 0 | 0 |
| *Salmonella enterica serovar Paratyphi C* RKS4594 | NC_012125.1 | Sa | 985289 | 993432 | 8143 | 0 | 0 | 0 | 0 | 0 | 0 | 0 | 0 |
| *Salmonella enterica serovar Paratyphi B* SPB7 | NC_010102.1 | Sa | 2130556 | 2138698 | 8142 | 0 | 0 | 0 | 0 | 0 | 0 | 0 | 0 |
| *Salmonella enterica serovar Gallinarum* SL287/91 | NC_011274.1 | Sa | 956425 | 964570 | 8145 | 0 | 0 | 0 | 0 | 0 | 0 | 0 | 0 |
| *Salmonella enterica serovar Enteritidis* P125109 | NC_011294.1 | Sa | 938525 | 946670 | 8145 | 0 | 0 | 0 | 0 | 0 | 0 | 0 | 0 |
| *Salmonella enterica serovar Dublin* CT_02021853 | NC_011205.1 | Sa | 1003758 | 1011644 | 7886 | 0 | 0 | 0 | 0 | 0 | 0 | 0 | 0 |
| *Salmonella enterica serovar Newport* SL254 | NC_011080.1 | Sa | 983956 | 992024 | 8068 | 0 | 0 | 0 | 0 | 0 | 0 | 0 | 0 |
| *Salmonella enterica Agona str.* SL483 | NC_011149.1 | Sa | 936369 | 946923 | 10554 | 0 | 0 | 0 | 0 | 0 | 0 | 0 | 0 |
| *Escherichia fergusonii* | NC_011740.1 | F | 1057075 | 1061657 | 4582 | 0 | 0 | 0 | 0 | 0 | 0 | 3 | 0 |
| *Escherichia coli* MG1655 | NC_000913.2 | A | 922136 | 926655 | 4519 | 0 | 0 | 0 | 0 | 0 | 0 | 2 | 0 |
| *Escherichia coli* W3110 | AC_000091.1 | A | 923335 | 927854 | 4519 | 0 | 0 | 0 | 0 | 0 | 0 | 2 | 0 |
| *Escherichia coli* DH10B | NC_010473.1 | A | 976064 | 980583 | 4519 | 0 | 0 | 0 | 0 | 0 | 0 | 2 | 0 |
| *Escherichia coli* BW2952 | NC_012759.1 | A | 825104 | 829623 | 4519 | 0 | 0 | 0 | 0 | 0 | 0 | 2 | 0 |
| *Escherichia coli* BL21(DE3) | NC_012947.1 | A | 2849897 | 2854769 | 4872 | 0 | 0 | 0 | 0 | 0 | 0 | 2 | 0 |
| Escherichia coli BL21 | NC_012892.1 | A | 927027 | 931899 | 4872 | 0 | 0 | 0 | 0 | 0 | 0 | 2 | 0 |
| *Escherichia coli* B-REL606 | NC_012967.1 | A | 939619 | 944490 | 4871 | 0 | 0 | 0 | 0 | 0 | 0 | 2 | 0 |
| *Escherichia coli* HS | NC_009800.1 | A | 983147 | 987780 | 4633 | 0 | 0 | 0 | 0 | 0 | 0 | 4 | 0 |
| *Escherichia coli* ATCC8739 | NC_010468.1 | A | 2982922 | 2988331 | 5409 | 0 | 0 | 0 | 0 | 0 | 0 | 4 | 0 |
| *Escherichia coli* IAI1 | NC_011741.1 | B1 | 974965 | 979543 | 4578 | 0 | 0 | 0 | 0 | 0 | 0 | 3 | 0 |
| *Escherichia coli* 55989 | NC_011748.1 | B1 | 984265 | 988783 | 4518 | 0 | 0 | 0 | 0 | 0 | 0 | 2 | 0 |
| *Escherichia coli* SE11 | NC_011415.1 | B1 | 1006787 | 1011306 | 4519 | 0 | 0 | 0 | 0 | 0 | 0 | 2 | 0 |
| *Escherichia coli* E24377A | NC_009801.1 | B1 | 972264 | 976842 | 4578 | 0 | 0 | 0 | 0 | 0 | 0 | 3 | 0 |
| *Shigella boydii* Sb227 | NC_007613.1 | Sh | 825717 | 830236 | 4519 | 0 | 0 | 0 | 0 | 0 | 0 | 2 | 0 |
| *Shigella boydii* CDC3083-94 | NC_010658.1 | Sh | 2246311 | 2250830 | 4519 | 0 | 0 | 0 | 0 | 0 | 0 | 2 | 0 |
| *Shigella sonnei* Ss046 | NC_007384.1 | Sh | 936670 | 941251 | 4581 | 0 | 0 | 0 | 0 | 0 | 0 | 3 | 0 |
| *Shigella flexneri 2a* Sf301 | NC_004337.1 | Sh | 874226 | 878804 | 4578 | 0 | 0 | 0 | 0 | 0 | 0 | 3 | 0 |
| *Shigella flexneri 2a* Sf2457T | NC_004741.1 | Sh | 869039 | 873617 | 4578 | 0 | 0 | 0 | 0 | 0 | 0 | 3 | 0 |
| *Shigella flexneri 2a* Sf8401 | NC_008258.1 | Sh | 908319 | 912897 | 4578 | 0 | 0 | 0 | 0 | 0 | 0 | 3 | 0 |
| *Shigella dysenteriae* Sd197 | NC_007606.1 | Sh | 2179234 | 2183751 | 4517 | 0 | 0 | 0 | 0 | 0 | 0 | 2 | 0 |
| *Escherichia coli* O157:H7 Sakai | NC_002695.1 | E | 1053988 | 1058509 | 4521 | 0 | 0 | 0 | 0 | 0 | 0 | 2 | 0 |
| *Escherichia coli* O157:H7 EDL933 | NC_002655.2 | E | 1055648 | 1147732 | 92084 | 0 | 0 | 0 | 0 | 0 | 0 | 2 | 0 |
| *Escherichia coli* O157:H7 EC4115 | NC_011353.1 | E | 1057344 | 1061865 | 4521 | 0 | 0 | 0 | 0 | 0 | 0 | 2 | 0 |
| *Escherichia coli* TW14359 | NC_013008.1 | E | 1058937 | 1063458 | 4521 | 0 | 0 | 0 | 0 | 0 | 0 | 2 | 0 |
| *Escherichia coli* UMN026 | NC_011751.1 | D | 1111560 | 1116555 | 4995 | 0 | 0 | 0 | 0 | 0 | 0 | 4 | 0 |
| *Escherichia coli* UTI89 | NC_007946.1 | B2 | 883698 | 897814 | 14116 | 1 | 1 | 1 | 1 | 1 | 1 | 9 | 7 |
| *Escherichia coli* APECO1 | NC_008563.1 | B2 | 885299 | 899235 | 13936 | 1 | 1 | 1 | 1 | 1 | 1 | 6 | 7 |
| *Escherichia coli* S88 | NC_011742.1 | B2 | 923728 | 937664 | 13936 | 1 | 1 | 1 | 1 | 1 | 1 | 6 | 7 |
| *Escherichia coli* CFT073 | NC_004431.1 | B2 | 977042 | 981561 | 4519 | 0 | 0 | 0 | 0 | 0 | 0 | 2 | 0 |
| *Escherichia coli* ED1a | NC_011745.1 | B2 | 879676 | 894744 | 15068 | 1 | 1 | 1 | 1 | 1 | 1 | 18 | 14 |
| *Escherichia coli* 536 | NC_008253.1 | B2 | 936646 | 941224 | 4578 | 0 | 0 | 0 | 0 | 0 | 0 | 3 | 0 |
| Escherichia coli O127:H6 E2348/69 | NC_011601.1 | B2 | 921191 | 925828 | 4637 | 0 | 0 | 0 | 0 | 0 | 0 | 1 | 0 |
| *Escherichia coli* IAI39 | NC_011750.1 | D | 2329745 | 2334506 | 4761 | 0 | 0 | 0 | 0 | 0 | 0 | 3 | 0 |
| *Escherichia coli* SMS35 | NC_010498.1 | D | 2259913 | 2305993 | 46080 | 0 | 0 | 0 | 0 | 0 | 0 | 1 | 0 |
