# Supplementary material for: The Small, Slow and Specialized CRISPR and Anti-CRISPR of Escherichia and Salmonella
Source: PLoS One. 2010 Jun 15;5(6):e11126. doi: 10.1371/journal.pone.0011126 (PMC2886076; doi:10.1371/journal.pone.0011126)
Supplement: Table S3 — The complete Ypest cas system is present in phylogenetically distant chromosomes and plasmids. (0.05 MB DOC) [file pone.0011126.s003.doc]

| ***Genome Sequences*** | ***Accession Number*** |
| --- | --- |
| *Acinetobacter baumannii* AYE | NC_010410.1 |
| *Acinetobacter baumannii* AB307-0294 | NC_011595.1 |
| *Acidovorax sp.* JS42 | NC_008782.1 |
| *Actinobacillus pleuropneumoniae* L20 | NC_009053.1 |
| *Actinobacillus pleuropneumoniae serovar* 3 str. JL03 | NC_010278.1 |
| *Actinobacillus pleuropneumoniae serovar* 7 str. AP76 | NC_010939.1 |
| *Chromobacterium violaceum* ATCC 12472 | NC_005085.1 |
| *Enterobacter* sp. 638 | NC_009436.1 |
| *Erwinia carotovora subsp. atroseptica* SCRI1043 | NC_004547.2 |
| *Erwinia tasmaniensis* Et1/99 | NC_010694.1 |
| *Escherichia coli* UTI89 | NC_007946.1 |
| *Escherichia coli* APEC O1 | NC_008563.1 |
| *Escherichia coli* ED1a | NC_011745.1 |
| *Escherichia coli* S88 | NC_011742.1 |
| *Legionella pneumophila* str. Lens plasmid pLPL | NC_006366.1 |
| *Pasteurella multocida subsp. multocida* str. Pm70 | NC_002663.1 |
| *Pseudomonas aeruginosa* UCBPP-PA14 | NC_008463.1 |
| *Shewanella baltica* OS195 | NC_009997.1 |
| *Shewanella baltica* OS185 | NC_009665.1 |
| *Shewanella* sp. W3-18-1 | NC_008750.1 |
| *Verminephrobacter eiseniae* EF01-2 | NC_008786.1 |
| *Yersinia pestis* CO92 | NC_003143.1 |
| *Yersinia pestis* KIM 10 | NC_004088.1 |
| *Yersinia pestis biovar Microtus* str. 91001 | NC_005810.1 |
| *Yersinia pestis Antiqua* | NC_008150.1 |
| *Yersinia pestis Nepal516* | NC_008149.1 |
| *Yersinia pestis Pestoides F* | NC_009381.1 |
| *Yersinia pestis Angola* | NC_010159.1 |
| *Yersinia pseudotuberculosis* IP 32953 | NC_006155.1 |
| *Yersinia pseudotuberculosis* IP 31758 | NC_009708.1 |
| *Yersinia pseudotuberculosis* YPIII | NC_010465.1 |
| *Yersinia pseudotuberculosis* PB1/+ | NC_010634.1 |
| *Zymomonas mobilis subsp. mobilis* ZM4 | NC_006526.1 |
| *Delftia acidovorans* SPH-1 | NC_010002.1 |
| *Legionella pneumophila* str. Lens | NC_006369.1 |
| *Coxiella burnetii* CbuG_Q212 | NC_011527.1 |
| *Coxiella burnetii Dugway* 5J108-111 | NC_009727.1 |
