# Supplementary material for: The Small, Slow and Specialized CRISPR and Anti-CRISPR of Escherichia and Salmonella
Source: PLoS One. 2010 Jun 15;5(6):e11126. doi: 10.1371/journal.pone.0011126 (PMC2886076; doi:10.1371/journal.pone.0011126)
Supplement: Table S4 — CRISPR1 and CRISPR2 match a small number of proto-spacer in the following known phages and plasmids. (0.03 MB DOC) [file pone.0011126.s004.doc]

| ***Accession number*** | ***Name*** | ***Type*** | ***Charactersitic*** | ***Family*** | ***Host*** |
| --- | --- | --- | --- | --- | --- |
| NC_002371 | Enterobacteria phage P22 | Virus | dsDNA  Podoviridae | P22-like | *S. enterica serovar typhimurium* |
| NC_004313 | Salmonella phage ST64B | Virus | dsDNA  Myoviridae | Na | *S. enterica subsp. Enterica serovar* |
| NC_004348 | Enterobacteria phage ST64T | Virus | dsDNA  Podoviridae | P22-like | *S. enterica subsp. Enterica serovar* |
| NC_005856 | Enterobacteria phage P1 | Virus | dsDNA  Myoviridae | P1-like | *E. coli* |
| NC_007804 | Enterobacteria phage phiV10 | Virus | dsDNA  Podoviridae | Epsilon15-like | *E.coli* O157:H7 |
| NC_010392 | Phage Gifsy-1 | Virus | dsDNA  Siphoviridae | Lambda-like | *S. typhimurium* LT2 |
| NC_013370 | Phage p0111_2 | Virus | dsDNA  Myoviridae | P1-like | E. col 0111:H- str. 11128 |
| NC_013365 | Plasmid p0111_1 | Plasmid | conjugatif |  | E. col 0111:H- str. 11128 |
| NC_002305 | Plasmid R27 | Plasmid | conjugatif |  | *S. typhimurium* LT2 |
| NC_009981 | Plasmid pMAK1 | Plasmid | conjugatif |  | *S. enterica subsp. Enterica serovar* |
| NC_003384 | Plasmid pHCM1 | Plasmid | conjugatif |  | *S. enterica Typhi* CT18 |
