# Supplementary material for: The Small, Slow and Specialized CRISPR and Anti-CRISPR of Escherichia and Salmonella
Source: PLoS One. 2010 Jun 15;5(6):e11126. doi: 10.1371/journal.pone.0011126 (PMC2886076; doi:10.1371/journal.pone.0011126)
Supplement: Table S5 — Conservation of the terminal repeat sequence of the CRISPR3 and CRISPR4. (0.08 MB DOC) [file pone.0011126.s005.doc]

| ***Genome Sequences*** | ***Phylogenetic-Group*** | ***terminal repeat sequence CRISPR3*** | ***termianl repeat sequence CRISPR4*** |
| --- | --- | --- | --- |
| *Escherichia fergusonii* | F | GTTCACTGCCGTACAGGCAGATAAAATG |  |
| *Escherichia coli* MG1655 | A | GTTCACTGCCGTACAGACAGATAAAATG |  |
| *Escherichia coli* W3110 | A | GTTCACTGCCGTACAGACAGATAAAATG |  |
| *Escherichia coli* DH10B | A | GTTCACTGCCGTACAGACAGATAAAATG |  |
| *Escherichia coli* BW2952 | A | GTTCACTGCCGTACAGACAGATAAAATG |  |
| *Escherichia coli* BL21(DE3) | A | GTTCACTGCCGTACAGACAGATAAAATG |  |
| Escherichia coli BL21 | A | GTTCACTGCCGTACAGACAGATAAAATG |  |
| *Escherichia coli* B-REL606 | A | GTTCACTGCCGTACAGACAGATAAAATG |  |
| *Escherichia coli* HS | A | GTTCACTGCCGTACAGACAGATAAAATG |  |
| *Escherichia coli* ATCC8739 | A | GTTCACTGCCGTACAGACAGATAAAATG |  |
| *Escherichia coli* IAI1 | B1 | GTTCACTGCCGTACAGACAGATAAAATG |  |
| *Escherichia coli* 55989 | B1 | GTTCACTGCCGTACAGACAGATAAAATG |  |
| *Escherichia coli* SE11 | B1 | GTTCACTGCCGTACAGACAGATAAAATG |  |
| *Escherichia coli* E24377A | B1 | GTTCACTGCCGTACAGACAGATAAAATG |  |
| *Shigella boydii* Sb227 | Sh | GTTCACTGCCGTACAGACAGATAAAATG |  |
| *Shigella boydii* CDC3083-94 | Sh | GTTCACTGCCGTACAGACAGATAAAATG |  |
| *Shigella sonnei* Ss046 | Sh | GTTCACTGCCGTACAGAAAGATAAAATG |  |
| *Shigella flexneri 2a* Sf301 | Sh | GTTCACTGCCGTACAGACAGATAAAATG |  |
| *Shigella flexneri 2a* Sf2457T | Sh | GTTCACTGCCGTACAGACAGATAAAATG |  |
| *Shigella flexneri 2a* Sf8401 | Sh | GTTCACTGCCGTACAGACAGATAAAATG |  |
| *Shigella dysenteriae* Sd197 | Sh | GTTCACTGCCGTACAGGCAGATAAAATG |  |
| *Escherichia coli* O157:H7 Sakai | E | GTTCACTGCCGTACAGGCAGATAAAATG |  |
| *Escherichia coli* O157:H7 EDL933 | E | GTTCACTGCCGTACAGGCAGATAAAATG |  |
| *Escherichia coli* O157:H7 EC4115 | E | GTTCACTGCCGTACAGGCAGATAAAATG |  |
| *Escherichia coli* TW14359 | E | GTTCACTGCCGTACAGGCAGATAAAATG |  |
| *Escherichia coli* UMN026 | D | GTTCACTGTCGAACAGGCAGATAAAATG |  |
| *Escherichia coli* UTI89 | B2 | GTTCACTGCCGTACAGGCAGTATTCACA | GTTCACTGCCGTACAGGCAGTCTTCAAA |
| *Escherichia coli* APECO1 | B2 | GTTCACTGCCGTACAGGCAGTATTCACA | GTTCACTGCCGTACAGGCAGTCTTCAAA |
| *Escherichia coli* S88 | B2 | GTTCACTGCCGTACAGGCAGTATTCACA | GTTCACTGCCGTACAGGCAGTCTTCAAA |
| *Escherichia coli* CFT073 | B2 | GTTCACTGCCGTACAGGCAGATAAAATG |  |
| *Escherichia coli* ED1a | B2 | CCTCACTGCCGTACAGGCAGTATTCACA | GTTCACTGCCGTACAGGCAGTCTTCAAA |
| *Escherichia coli* 536 | B2 | GTTCACTGCCGTACAGGCAGATAAAATG |  |
| Escherichia coli O127:H6 E2348/69 | B2 | GTTCACTGCCGTACAGGCAGATAAAATG |  |
| *Escherichia coli* IAI39 | D | GTTCACTGCCGTACAGGCAGATAAAATG |  |
| *Escherichia coli* SMS35 | D | GCTCACTGCCGTACAGGCAGATAAAATG |  |
