# Supplementary material for: The Small, Slow and Specialized CRISPR and Anti-CRISPR of Escherichia and Salmonella
Source: PLoS One. 2010 Jun 15;5(6):e11126. doi: 10.1371/journal.pone.0011126 (PMC2886076; doi:10.1371/journal.pone.0011126)
Supplement: Table S6 — Example of stretches of conserved repeat variants among the CRISPR array. (0.04 MB DOC) [file pone.0011126.s006.doc]

**Table S6:** Example of stretches of conserved repeat variants among the CRISPR array

| *Salmonella enterica serovar Schwarzengrund* CVM19633 - NC_011094.1 | | | | |
| --- | --- | --- | --- | --- |
|  | ***Start*** | ***End*** | ***Mismatch*** | ***Repeat-Sequence*** |
| 1 | 2999469 | 2999497 | 5 | ACGGCTATCCCCGCTGACGCGGGGAACAC |
| 2 | 2999530 | 2999558 | 1 | CGGTTTATCCCCGCTAGCGCGGGGAACAC |
| 3 | 2999591 | 2999619 | 1 | CGGTTTATCCCCGCTGACGCGGGGAACAC |
| 4 | 2999652 | 2999680 | 1 | CGGTTTATCCCCGCTGACGCGGGGAACAC |
| 5 | 2999713 | 2999741 | 1 | CGGTTTATCCCCGCTGACGCGGGGAACAC |
| 6 | 2999774 | 2999802 | 0 | CGGTTTATCCCCGCTGGCGCGGGGAACAC |
| 7 | 2999835 | 2999863 | 0 | CGGTTTATCCCCGCTGGCGCGGGGAACAC |
| 8 | 2999896 | 2999924 | 0 | CGGTTTATCCCCGCTGGCGCGGGGAACAC |
| 9 | 2999957 | 2999985 | 0 | CGGTTTATCCCCGCTGGCGCGGGGAACAC |
| 10 | 3000018 | 3000046 | 0 | CGGTTTATCCCCGCTGGCGCGGGGAACAC |
| 11 | 3000079 | 3000107 | 1 | CGGTTTATCCCCGCTGGCGCGGGGAATAC |
| 12 | 3000140 | 3000168 | 1 | CGGTTTATCCCCGCTGGCGCGGGGAATAC |
| 13 | 3000201 | 3000229 | 1 | CGGTTTATCCCCGCTGGCGCGGGGAATAC |
| 14 | 3000262 | 3000290 | 1 | CGGTTTATCCCCGCTGGCGCGGGGAATAC |
| 15 | 3000323 | 3000351 | 1 | CGGTTTATCCCCGCTGGCGCGGGGAATAC |
| 16 | 3000384 | 3000412 | 1 | CGGTTTATCCCCGCTGGCGCGGGGAATAC |
| 17 | 3000445 | 3000473 | 1 | CGGTTTATCCCCGCTGGCGCGGGGAATAC |
| 18 | 3000506 | 3000534 | 1 | CGGTTTATCCCCGCTGGCGCGGGGAATAC |
